# Opto-acoustic synergistic irradiation for vaporization of natural melanin-cored nanodroplets at safe energy levels and efficient sono-chemo-photothermal cancer therapy

**DOI:** 10.7150/thno.44879

**Published:** 2020-08-20

**Authors:** Yaxin Hu, Shan Xue, Ting Long, Peizhao Lyu, Xinyu Zhang, Jingqin Chen, Siping Chen, Chengbo Liu, Xin Chen

**Affiliations:** 1 National-Regional Key Technology Engineering Laboratory for Medical Ultrasound, School of Biomedical Engineering, Health Science Center, Shenzhen University, Shenzhen, 518060, P.R. China.; 2 Research Laboratory for Biomedical Optics and Molecular Imaging, CAS Key Laboratory of Health Informatics, Shenzhen Institutes of Advanced Technology, Chinese Academy of Sciences, Shenzhen 518055, China.

**Keywords:** perfluoropentane nanodroplets, melanin, doxorubicin, synergistic irradiation, cancer therapy

## Abstract

**Rationale:** Insufficient penetration and accumulation of theranostic payloads in solid tumors greatly challenge the clinical translation of cancer nanomedicines. To address this challenge, we synthesized natural melanin-cored and doxorubicin-loaded perfluoropentane nanodroplets with good biocompatibility and self-assembling ability.

**Methods:** We used an opto-acoustic synergistic irradiation (OASI) method that was effective at lower energy levels than ultrasound- or laser-only irradiation to safely vaporize the nanodroplets and to cavitate the generated microbubbles for mechanically enhancing intratumoral delivery. The delivered melanin and doxorubicin inside the tumors mediated secondary chemo-photothermal therapy under laser irradiation to fully kill cancer cells.

**Results:** *In vivo* animal experiments demonstrated direct mechanical disruption of tumor structures (H&E staining), enhanced intratumoral penetration of melanin (photoacoustic imaging), and efficient intratumoral accumulation of doxorubicin (fluorescent imaging). Anti-tumor experiments demonstrated that the nanodroplets combined with OASI treatment and subsequent laser irradiation could efficiently eliminate melanoma tumors.

**Conclusion:** Melanin-cored and doxorubicin-loaded perfluoropentane nanodroplets hold great promise for translational sono-chemo-photothermal cancer therapy.

## Introduction

Considerable success has been achieved in the development of functional nanoparticles for conducting chemo-, gene-, immuno-, and photothermal cancer therapy. Nanoparticles, such as liposome-carrying chemotherapeutics and iron oxide nanoparticles that mediate hyperthermia therapy, have been clinically approved for treating patients suffering from leukemia and glioblastoma, respectively 1, 2. In addition to cancer therapy, nanoparticles also have great potential in the molecular diagnosis of early-stage cancers 3. The efficacy of nanoparticle-based cancer therapy or diagnosis positively correlates with the nanoparticle concentration in the tumor region 4. Therefore, it is imperative that nanoparticles can efficiently penetrate and accumulate in tumor regions for cancer theranostics. Although tumor vessels are leaky and the lymphatic drainage of tumors is defective owing to the enhanced permeability and retention (EPR) effect 5, deep and uniform penetration of nanoparticles is still challenging because of the complexity and heterogeneity of solid tumors. For example, the total concentration of nanoparticles accumulated by the EPR effect has been shown to vary substantially among tumors of different patients, and among tumors of different type and size 6. Even for the same tumor, the permeability of blood vessels for nanoparticle extravasation varies from segment to segment 7.

Phase‐change microdroplets and nanodroplets have been used to mechanically disrupt intratumoral barriers to enhance nanoparticle penetration and accumulation. Upon stimulation (e.g., heating), the droplets can be vaporized into gaseous bubbles owing to the low boiling point of their perfluorocarbon cores (e.g., 29 °C for perfluoropentane (PFP)). The diameter of these bubbles is approximately 4-5 times larger than that of the droplets 8. We note that the mechanical forces generated during bubble growth can damage the surrounding endothelial cells and increase the permeability of blood vessels in tumor regions 9, 10. In particular, when ultrasound is used to acoustically trigger the phase transition of nanodroplets, the microbubbles that are produced can undergo cavitation, either in the form of stable oscillations or inertial collapse to induce violent physical events (i.e., fluid streaming and jetting) 11, 12. In this way, the mechanical effects induced by nanodroplet vaporization and microbubble cavitation not only increase nanoparticle extravasation (2.3-fold), but also enhance nanoparticle penetration (to a depth of >70 µm) 13. However, when ultrasound (at peak negative pressures of 8.6-20 MPa) is used alone for nanodroplet vaporization, the calculated mechanical index of the sound waves far exceeds the US Food and Drug Administration (FDA) safety limit of 1.9 14, 15.

When photothermal agents (e.g., gold nanoparticles) are included in the perfluorocarbon core of the nanodroplets, laser irradiation can also be used for droplet vaporization 16, 17. For example, when irradiated by an 808-nm laser at 1 W/cm^2^ for 5 minutes, gold nanorods with concentrations of 5-80 ppm increased the temperature of the surrounding medium by 14-43 °C, which efficiently triggered nanoparticle vaporization for cancer theranostics 18. However, the clinical translation of gold nanoparticles for nanodroplet vaporization is hindered by their poor biocompatibility 19. To overcome this challenge, we turned our attention to melanin, a naturally-existing pigment with intrinsic biocompatibility, which has demonstrated good photothermal conversion ability and has exhibited great promise in photothermal cancer therapy and drug release 20, 21. In this work, we propose a new nanodroplet platform that uses natural melanin as the photothermal core for nanodroplet vaporization.

The challenges for vaporizing natural melanin-cored nanodroplet to increase tumor permeability are: (i) the surface tension of the nanodroplet shell increases the boiling point of the liquid PFP core by 40 °C 22; and (ii) the photothermal transition efficiency of melanin is lower than that of gold nanoparticles, which exhibit the surface plasmon resonance effect. These two challenges would necessitate high laser intensities for nanodroplet vaporization that exceed safety levels for *in vivo* irradiation (i.e., >1 W/cm^2^). To overcome this challenge, we used an opto-acoustic synergistic irradiation (OASI) method, which has lower total energy than either ultrasound or laser irradiation alone, to safely vaporize the nanodroplets. Using gold nanospheres as the photothermal agent to decorate the nanodroplets, Arnal et al. demonstrated that the combination of ultrasound exposure and laser heating increased the efficiency of nanodroplet vaporization for sono-photoacoustic imaging 23. In our approach, the introduction of ultrasound not only decreases the energy threshold of laser irradiation for vaporization of melanin-cored nanodroplets, but also triggers the cavitation of the microbubbles generated by vaporization to increase melanin penetration in tumor regions, increasing the efficacy of photothermal cancer therapy.

We used polyvinyl alcohol (PVA), which is non‐toxic, biodegradable, and highly hydrophilic, as the shell material of the nanodroplets 24. Considering that liquid PFP has very low solubility in water and good solubility in ethanol, we used the Ouzo effect to spontaneously generate PVA‐shelled and melanin‐cored nanodroplets by directly adding melanin-PFP-ethanol solution into the PVA-water solution without using any harmful surfactant or solvent 25. Moreover, to fully kill the cancer cells, we further loaded the anti‐cancer drug doxorubicin (DOX) onto the PVA shells of the nanodroplets because multimodal cancer therapy is more effective than monomodal therapies 26. We hypothesize that after nanodroplet vaporization and microbubble cavitation, the loaded DOX would also be released into the surrounding tumor microenvironment because of shell rupture. This should allow the melanin and DOX to accumulate in the tumors to facilitate secondary chemo-photothermal therapy upon laser irradiation. In this way, we synergistically combined the mechanical wounding effects of ultrasound contrast agents, the cytotoxic effects of a chemotherapy drug, and the photothermal effects of melanin for sono-chemo-photothermal cancer therapy. The *in vitro* and *in vivo* anti-tumor efficiency of this synergistic method was examined using a murine melanoma tumor model.

## Materials and Methods

### Extraction of melanin nanoparticles

Fresh cuttlefish were purchased from a local market (Dasheng, Shenzhen, China). First, the ink sac of the cuttlefish was dissected and the extracted ink was diluted in distilled water at a volume ratio of 1:9. To fully disperse the melanin nanoparticles, the ink solution was ultrasonicated at 50 W (50% duty cycle) for 30 minutes. Large particles inside the ink solution were removed using 25-mm-diameter syringe filters with a 1-µm pore size. Then, the melanin nanoparticles were extracted by centrifugation (12,000 rpm) at 4 °C for 20 minutes and washed with distilled water three times. The extracted melanin nanoparticles were suspended in distilled water and freeze‐dried. The melanin powder was stored at -20 °C.

### Nanodroplet synthesis

The melanin‐cored nanodroplets were synthesized based on the Ouzo effect. The organic-phase solution was prepared in two steps: 40 mg melanin powder was first dissolved in 1 mL ethanol by ultrasonication at 80 W power (50% duty cycle) for 5 minutes, and then the melanin-in-ethanol solution was mixed with 200 µL PFP (perfluoropentane, C_5_F_12_, CAS: 678-26-2, Strem Chemicals, Inc., Newburyport, MA, USA) by agitation. As shown in Supplementary Figure 1, PFP fully dissolved in the ethanol solution containing melanin nanoparticles. Although the organic-phase solution was a well-mixed homogenous system, we found that melanin nanoparticles gradually sedimented. Therefore, this solution was used for nanodroplet synthesis immediately after mixing. We also note that the PFP did not fully dissolve in ethanol when its volume ratio was greater than or equal to 20% (Supplementary Figure 2).

The aqueous-phase solution was prepared by dissolving 0.1 g PVA (polyvinyl alcohol, 31 kDa, CAS: 9002-89-5, Sigma-Aldrich, Corp., St Louis, MO, USA) in 20 mL distilled water at 95 °C for 30 minutes. To create the nanodroplet emulsion, 0.5 mL organic-phase solution was added to 1.5 mL aqueous-phase solution (PVA concentration of 0.5% w/w) and fully mixed by pipetting. In this way, the solubility of PFP decreased. Therefore, PFP and its embedded melanin underwent spontaneous emulsification and formed PVA-shelled and melanin-cored nanodroplets. To further illustrate the emulsification process, we removed melanin nanoparticles from the organic phase, and added the mixture of PFP and ethanol directly to the aqueous phase. As shown in Supplementary Figure 3A, spontaneous emulsifycation occurred after pipetting and the formation of nanodroplets resulted in a milky solution. By contrast, no formation of nanodroplets was observed when only ethanol was added, and the solution remained clear (Supplementary Figure 3B). The calculated final concentration of melanin in the nanodroplet emulsion was 10 mg/mL. To remove the excess PVA and the ethanol, dialysis was performed on the nanodroplet emulsion using a dialysis bag (MWCO 100 kDa) at 4 °C for 6 hours.

**Figure 1:**
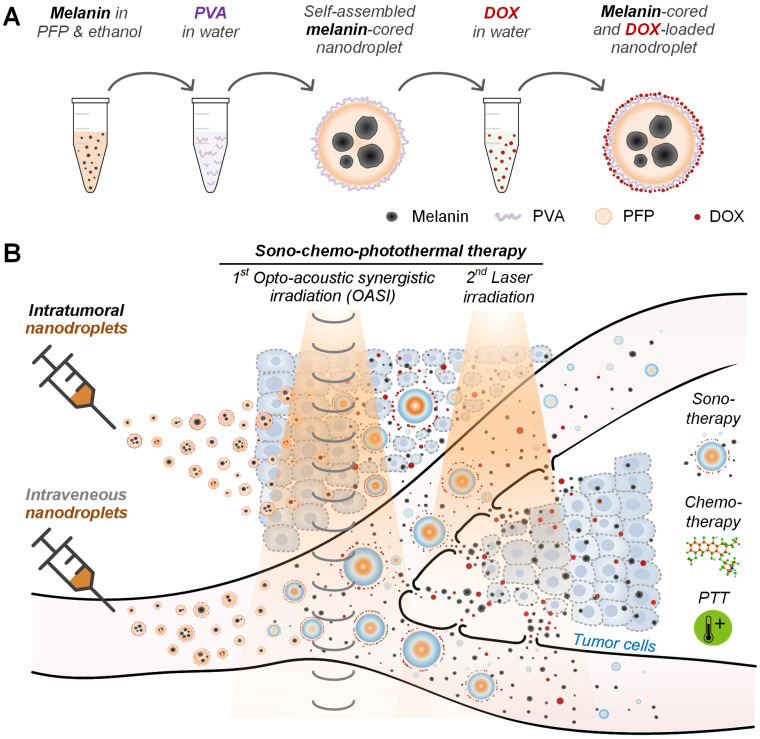
Schematic illustration of (**A**) the synthesis of the self-assembled, natural melanin-cored and doxorubicin-loaded nanodroplets, and (**B**) sono-chemo-photothermal therapy conducted in two steps (OASI followed by laser irradiation) and mediated by either intravenously- or intratumorally-injected nanodroplets. DOX: doxorubicin. PTT: photothermal therapy.

To incorporate a chemo-therapeutic function into the melanin-cored nanodroplets, the anti-cancer drug, DOX (doxorubicin hydrochloride, CAS: 25316-40-9, Sigma-Aldrich, Corp., St Louis, MO, USA), was added into the dialyzed emulsion at different final concentrations (0.1, 0.5, and 2.5 mM). It has been reported that DOX is cationic 27. Therefore, we were able to load positively-charged DOX onto the negatively-charged PVA shells of the nanodroplets via electrostatic interactions 28, 29. The emulsion was stored at 4 °C for 24 hours to allow full incorporation of DOX into the PVA shells. To remove free DOX from the nanodroplet solution, dialysis was performed for a second time using the method described above. The drug-loading efficiency (DLE) was calculated according to the following formula: DLE (wt%) = (weight of the loaded drug/weight of the feeding drug) × 100%. The synthesis process of the DOX‐loaded, PVA‐shelled, and melanin‐cored nanodroplet is illustrated in Figure 1A.

### Nanodroplet characterization

The morphology of the melanin nanoparticles and the DOX-loaded, melanin-cored nanodroplets was imaged using a transmission electron microscope (TEM) (HT7700; Hitachi, Tokyo, Japan) at 80 kV and a scanning electron microscope (SEM) (SU-70, Hitachi, Tokyo, Japan) at 5.0 kV. The size distribution and the zeta potential of the nanoparticles and droplets were characterized using a Zetasizer (Nano-ZS90, Malvern Instruments, Worcestershire, UK). Absorption spectroscopy of the nanodroplets loaded with and without DOX in the ultraviolet and visible spectral range was performed using a spectrophotometer (Cary 60, Agilent Technologies, Santa Clara, CA, USA).

### Nanodroplet vaporization by heating or laser irradiation

To examine the vaporization of the DOX-loaded, melanin-cored nanodroplets, the nanodroplet solution was diluted (1:2) and placed in 60 °C water for 2 minutes. To investigate the vaporization efficiency of the nanodroplets under different laser-irradiation conditions, 10 µL diluted nanodroplet solution was pipetted into the microwells of 35-mm glass-bottom dishes (P35G-0-10-C, Mat-Tek Corp., Ashland, MA, USA), and the solution was evenly spread by placing a 12-mm-diameter round cover glass onto the microwells. The energy spot of an 808-nm laser (cat. no.: LWIRL808, Laser Wave Corp., Beijing, China) was aligned with the center of the imaging view of an inverted microscope (Axio Observer A1, Carl Zeiss, Inc., Oberkochen, Germany). Various laser intensities (1, 2, 3, 4 W/cm^2^) were calibrated using a laser power meter (VLP-2000, Ranbond, Beijing, China). Laser-induced vaporization of the nanodroplets was observed in real time under the inverted microscope.

### Nanodroplet vaporization by combined ultrasound and laser irradiation

The pulsed ultrasound wave was edited in a function generator (DG1022, Agilent Technologies, Palo Alto, CA, USA), amplified by a power amplifier (2100L, Electronics and Innovation Ltd., Rochester, NY, USA), and produced by a high-intensity focused ultrasound (HIFU) transducer (H102; Sonic Concepts, Bothell, WA, USA). To vaporize the nanodroplets using the synergistic effect of photothermal heating and ultrasound cavitation, the focal region of the ultrasound (-6-dB profile of 9.97-mm length and 1.34-mm width) was confocally-aligned with that of the laser (full width at half maximum of 12 mm). A silicone tube (inner diameter of 1.5 mm and outer diameter of 3.5 mm) containing the diluted nanodroplet solution (1:2) was placed at the confocal region of the ultrasound and the laser. To visualize the generation and cavitation of microbubbles, a commercial ultrasound imaging system (SonixTouch, Ultrasonix Medical Corp., Richmond, BC, Canada) was used with optimized imaging parameters (frequency of 14.0 MHz, imaging depth of 5.0 cm, and dynamic range of 70 dB). The acoustic parameters of the ultrasound generated in the focal region were measured with a hydrophone (HNR-0500, ONDA Corp., USA). To avoid *in vivo* tissue damage, the laser power was fixed at 1 W/cm^2^. Pulsed ultrasound (1.1-MHz frequency, 5-cycle pulse duration, and 1-Hz pulse repetition frequency) of different peak negative pressures (0.20, 0.41, 0.62, 0.82, 1.00 MPa) was applied simultaneously with laser irradiation. The spatial-peak temporal-average intensity (*I*_SPTA_) of the pulsed ultrasound was calculated according to the well-established formula 30.

### Cell culture and cytotoxicity assessment

Human melanoma cells (A375, ATCC® CRL-1619™) were cultured in Dulbecco's modified Eagle's Medium (DMEM) supplemented with 10% fetal bovine serum (FBS). After reaching a 70% confluence in 25 cm^2^ flasks, the cells were rinsed with phosphate buffered saline (PBS) and detached using 0.25% trypsin-EDTA solution. Then, the cells were seeded at a density of 1.5 ×10^4^ cells per well into a 96-well plate and cultured for 24 hours. To investigate the photothermal therapeutic efficacy, nanodroplets of various melanin concentrations (1.25-10 mg/mL) without DOX loading were added into the cell culture medium. The wells containing the cells and the nanodroplets were then exposed to 808-nm laser irradiation at different intensities (1-3 W/cm^2^) for 5 minutes. To investigate the chemotherapeutic efficacy, nanodroplets of various DOX loading concentrations (13.8-110 µM) were added into the cell culture medium. To examine the synergistic effect of photothermal therapy and chemotherapy, the cells were incubated with DOX-loaded nanodroplets and laser-irradiated for 5 minutes.

The cell viability was examined 24 hours after each treatment using the CCK-8 colorimetric assay. Briefly, for a well containing 200 µL culture medium, 20 µL CCK-8 solution was added. After a 2-hour incubation period, the absorbance of the culture medium at 450 nm was measured using a microplate reader (Synergy H1, BioTek, Winooski, VT, USA). To visualize the cells impaired by chemo-photothermal therapy, their cell nuclei were stained by the red fluorescence dye propidium iodide at a final concentration of 50 µg/mL for 15 minutes. The nuclei of the live cells were stained by the blue fluorescence dye Hoechst 33342 at a concentration of 10 µg/mL for 15 minutes. Then, the labelled nuclei were imaged using a confocal microscope (C2 Plus, Nikon, Tokyo, Japan). All cell culture materials and fluorescent dyes were purchased from Thermo Fisher Scientific (Waltham, MA, USA).

### Animals and xenograft tumor model

Female 3-week-old athymic BALB/c nude mice were purchased from Guangdong Medicinal Laboratory Animal Center (Guangzhou, Guangdong, China). The animal experiments were approved by the Animal Ethical and Welfare Committee of Health Science Center of Shenzhen University. To induce the formation of melanoma tumors, 3 × 10^6^ A375 cells in 100 µL PBS were subcutaneously injected into the hind leg of the mice at 4 weeks of age. The length and width of the tumors were measured daily using a digital Vernier caliper. The volume of the tumors was calculated using the formula 0.5 × (length) × (width^2^).

### *In vivo* photoacoustic imaging and temperature measurements

To examine the accumulation efficiency of melanin in the tumor regions, photoacoustic imaging and ultrasound imaging were performed at designated time points (6, 12, 24, 36 hours) after OASI treatment using a Vevo 2100 LAZR system (VisualSonics, Inc., New York, NY, USA). The center frequency of the ultrasound imaging transducer was 40 MHz and the wavelength of the excitation laser was 685 nm. 3D scanning of the whole tumor region was carried out at a scan distance of 10 mm and a step size of 0.2 mm. The tumor regions in both intravenous and intratumoral injection groups were monitored during laser irradiation (1 W/cm^2^) using a Fluke-Ti25 infrared thermal camera.

### Fluorescence imaging of DOX

To investigate the cellular uptake and distribution of DOX, A375 cells were seeded into a glass-bottom confocal dish at a density of 5 × 10^4^ cells per dish. Then, nanodroplets were diluted in CO_2_-independent medium (18045088, Gibco, Grand Island, NY, USA) at a volume ratio of 1:9. Immediately after the addition of the nanodroplets (-OASI group) or OASI treatment (+OASI group), the cells were imaged using confocal microscopy with an excitation wavelength of 488 nm and an emission filter of 560 nm. The live cells were imaged for 2 hours and the temperature was maintained at 37 °C.

To further examine the DOX distribution in the tumor slides, tumor tissues were dissected 24 hours after the addition of the nanodroplets (-OASI group) or OASI treatment (+OASI group). After washing three times with PBS buffer, the tumor tissues were embedded into optimal cutting temperature compound and frozen in liquid nitrogen. Then, the tissue was cut into 10 thick sections using a cryostat. Fluorescence images of the tumor slides were taken using the confocal microscope (488-nm excitation and 560-nm emission wavelengths).

For *ex vivo* fluorescence imaging of the DOX distribution in mice, the animals were sacrificed 24 hours after intravenous nanodroplet injection and OASI treatment. The major organs (i.e., heart, liver, spleen, lung, kidney, and tumor) were excised and washed three times with ice-cold PBS. The DOX fluorescence intensities in all organs were imaged and measured using a small-animal imaging system (IVIS Spectrum, Caliper Life Sciences, Hopkinton, MA, USA). Fluorescence imaging of DOX was performed using 475-nm excitation and 600-nm emission filters.

### *In vivo* sono-chemo-photothermal therapy

Before sono-chemo-photothermal therapy, 200 µL nanodroplet solution (10 mg/mL melanin and 110 μg/mL DOX) was injected either intravenously or intratumorally. Then, as illustrated in Figure 1B, sono-chemo-photothermal therapy of tumors (volume of ~80 mm^3^) was carried out in two steps: (i) OASI treatment using the laser (1 W/cm^2^) and ultrasound (1.1-MHz frequency, 0.82-MPa peak negative pressure, 5-cycle pulse duration, and 1-Hz pulse repetition frequency) for 2 minutes to mechanically disrupt blood vessels and cell membranes to increase the release of melanin and DOX into the tumors; and (ii) laser treatment (1 W/cm^2^) for 5 minutes to synergistically kill the tumor cells through the chemotherapeutic effect of DOX and the photothermal effect of melanin. The energy focus of the OASI was moved such that the whole tumor region could be irradiated. For the intravenous nanodroplet group, laser treatment was carried out 24 hours after OASI treatment; for the intratumoral nanodroplet group, laser treatment was carried out 5 minutes after OASI treatment. The size of the tumor and the weight of the mice were measured daily. Two weeks after the first round of sono-chemo-photothermal therapy, the mice were sacrificed and their major organs were excised for hematoxylin and eosin (H&E) staining.

### Pharmacokinetics of DOX in blood

Blood samples of 20 µL were collected from the tail vein of the mice by incision at 0.5, 1, 2, 4, and 6 hours after nanodroplet injection or OASI treatment. The blood was diluted in PBS buffer at a volume ratio of 1:1. Then, the samples were centrifuged at 5000 rpm for 5 minutes and 20 µL supernatant was transferred to a 384-well plate. The amount of DOX in the blood samples was quantified by measuring the fluorescence of DOX (excitation at 488 nm, emission at 560 nm) using a Tecan Infinite 200 PRO plate reader (Tecan Systems, San Jose, CA, USA).

### *In vivo* toxicity study of the nanodroplets

With the removal of an eyeball, ~1.2 mL blood was collected from the isoflurane-anesthetized mice. First, 0.2 mL blood was mixed with 10 µL EDTA solution (0.05 M) to prevent coagulation. The blood cells were counted using an automatic hematology analyzer (Hemaray 86, Rayto inc., Shenzhen, China). Then, 1.0 mL blood was placed at room temperature for 2 hours and centrifugated at 3000 rpm for 20 minutes to collect the serum. Biochemical analysis of the serum was carried out using an automatic biochemistry analyzer (Chemray 800, Rayto inc., Shenzhen, China).

## Results

### Characterization of the natural melanin-cored and DOX-loaded nanodroplets

**Figure 2:**
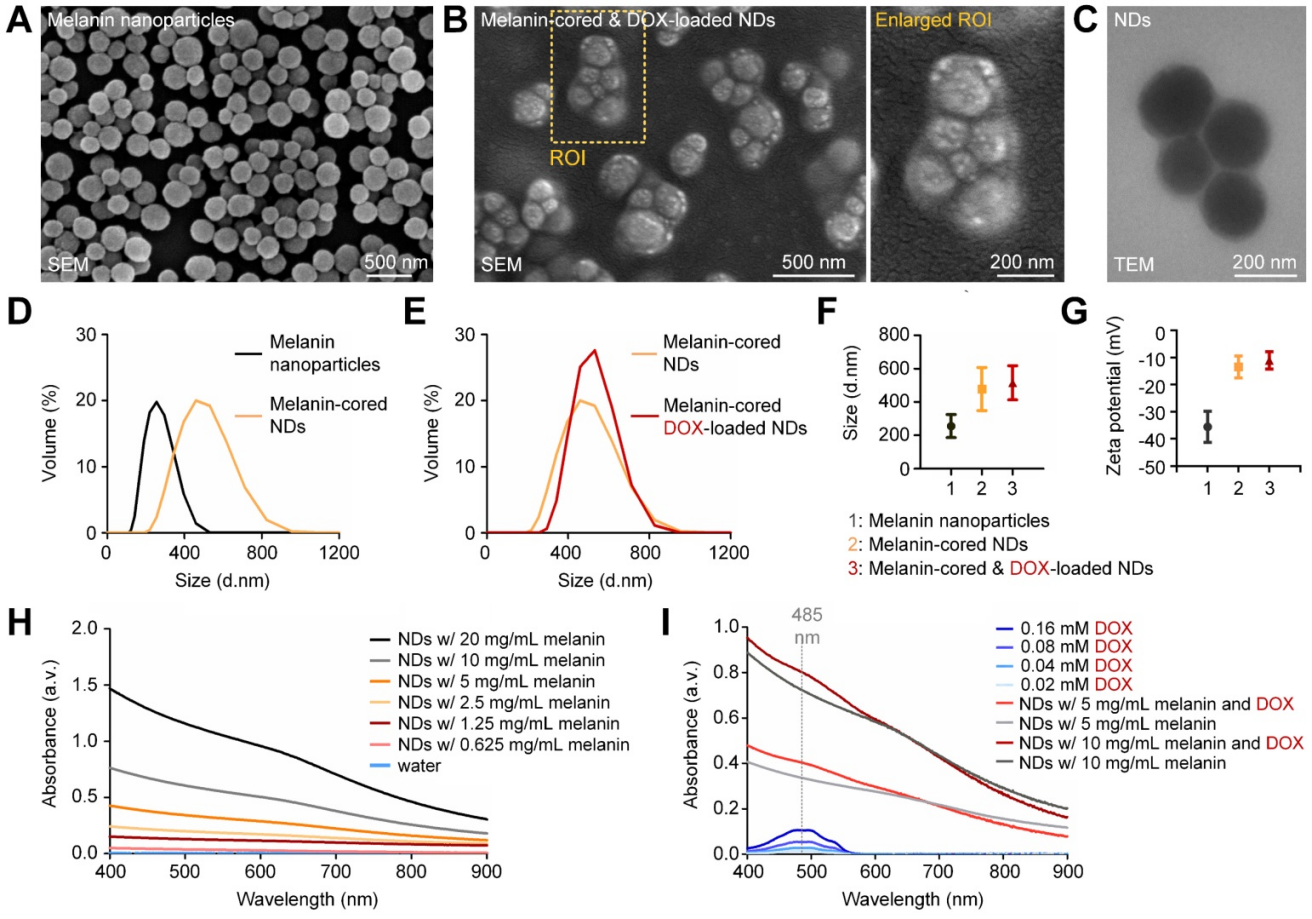
** Characterization of the natural melanin-cored and doxorubicin-loaded nanodroplets.** (**A**) SEM image of the melanin nanoparticles extracted from cuttlefish. (**B**) SEM image and (**C**) TEM image, which confirm that melanin is the core of the nanodroplets. (**D,E**) Size distribution and (**F,G**) zeta potential measurements of melanin nanoparticles, melanin-cored nanodroplets, and melanin-cored, DOX-loaded nanodroplets. (**H,I**) Absorption spectra of melanin-cored nanodroplets before and after DOX loading, respectively. NDs: nanodroplets. DOX: doxorubicin.

As shown in the SEM image in Figure 2A, the natural melanin nanoparticles had a spherical morphology and were well dispersed. After spontaneous emulsification by the Ouzo effect, 2-6 melanin nanoparticles and their surrounding PFP were encapsulated by PVA membranes (Figure 2B; enlarged image). The PVA-shelled and melanin-cored structure of the nanodroplets is also confirmed by the TEM image in Figure 2C. Quantitative analysis confirms that the nanodroplets have larger diameters and a broader size distribution than the melanin nanoparticles (Figure 2D). The loading of DOX onto the PVA shells of the nanodroplets had little influence on their size distribution (Figure 2E). The average diameters of the melanin nanoparticles, nanodroplets, and DOX-loaded nanodroplets were calculated to be 254.8 ± 69.53 nm, 477.6 ± 129.50 nm, and 515.2 ± 102.50 nm, respectively. The polydispersity index of the DOX-loaded nanodroplets was 0.21. The zeta potential of the melanin nanoparticles was -35.6 ± 5.77 mV and that of the nanodroplets was -13.0 ± 4.04 mV. When DOX was loaded onto the PVA shell, the zeta potential increased to -1.0 ± 3.42 mV. As shown in Supplementary Figure 4A, the zeta potential of the nanodroplets increased with the loaded amount of DOX.

As shown in Figure 2H, the optical absorption of the melanin-cored nanodroplets (without DOX loading) decreased with increasing wavelength, which is in line with the previously reported UV-visible spectrum of melanin. When the concentration of melanin in the nanodroplets decreased from 20 to 1.25 mg/mL, the absorption at 808 nm decreased from 0.436 to 0.082. The UV-visible spectra of DOX solution of various concentrations (0.02, 0.04, 0.08, 0.16 mM) were also measured (Figure 2I). The optical absorption peaked at 485 nm. The peak absorption coefficients of 0.08 and 0.16 mM DOX were 0.053 and 0.105, respectively. In contrast with the spectra without DOX loading, those with DOX loading had an obvious increase in absorption at 485 nm, confirming that DOX had been successfully attached to the PVA shells. The average absorption increase of the DOX-loaded nanodroplets at 485 nm was 0.072; therefore, the calculated concentration of DOX loaded onto the nanodroplets was 110 µM. The drug-loading efficiency was calculated to be 22%.

To investigate the loading capacity of DOX onto the PVA shell of the nanodroplets, we incubated the nanodroplets with DOX at a relatively high concentration of 2.5 mM. After the removal of free DOX by dialysis, the average absorption of DOX-loaded nanodroplets increased substantially at 485 nm (Supplementary Figure 4B). The calculated concentration of the loaded DOX was 366 µM. However, aggregation of nanodroplets and leakage of DOX were observed 6 hours after the dialysis of free DOX, which suggested that the synthesized nanodroplets were not stable (Supplementary Figure 4C; red arrow). Therefore, we used nanodroplets loaded with 110 µM DOX for sono-chemo-photothermal therapy. We note that the size of these nanodroplets gradually decreased during the 5-day observation period. However, there was no obvious difference in the stability of the nanodroplets observed when they were incubated in PBS buffer, DMEM medium, or DMEM medium supplemented with 10% FBS (Supplementary Figure 5).

### Nanodroplets vaporized by either heating or high-intensity laser irradiation

**Figure 3:**
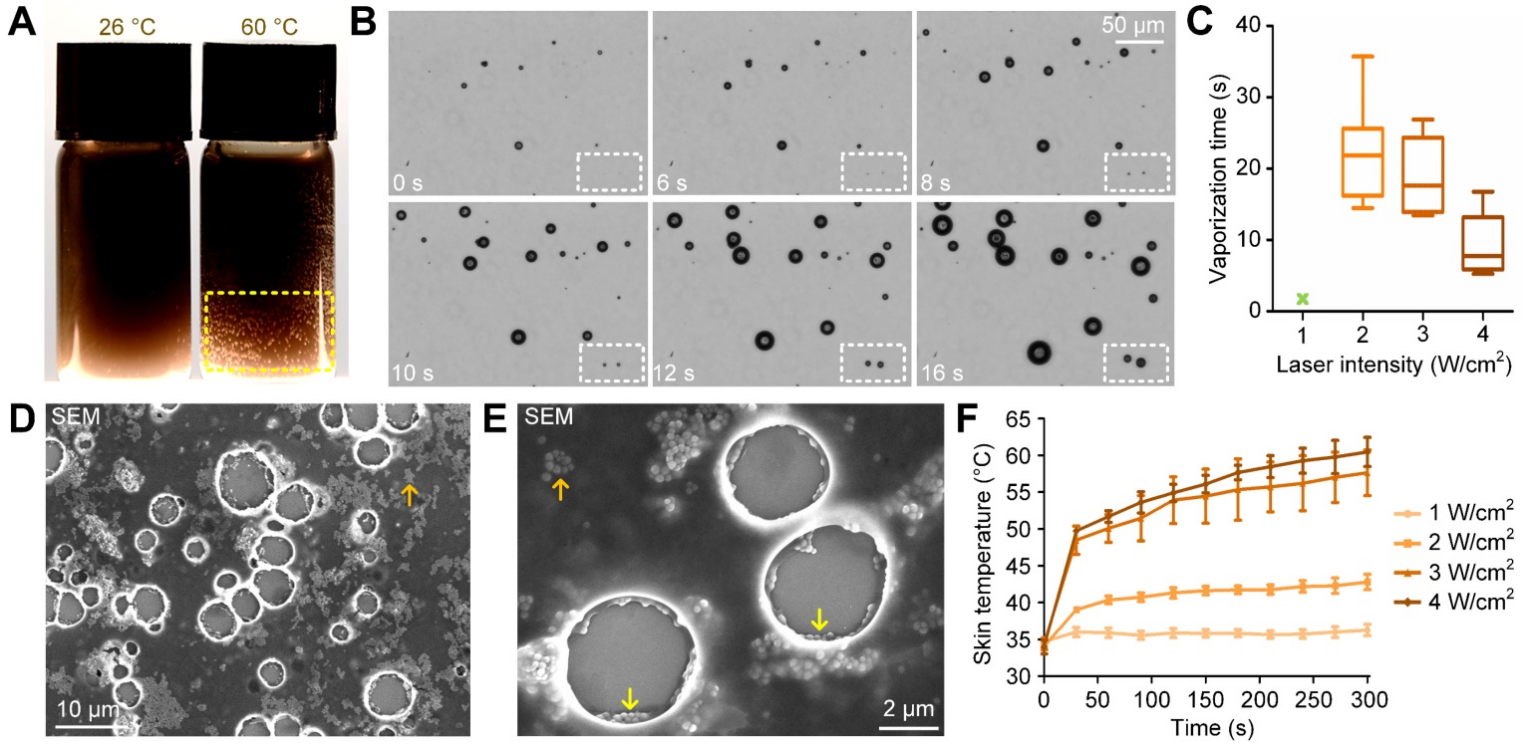
** Nanodroplets vaporized by either heating or high-intensity laser irradiation.** (**A**) Vaporization of nanodroplets after heating in 60 °C water for 2 minutes. (**B**) Time-lapsed microscope images showing the vaporization of the nanodroplets irradiated by the laser at 3 W/cm^2^. (**C**) Calculated vaporization time of nanodroplets irradiated at various laser intensities (1, 2, 3, 4 W/cm^2^). (**D,E**) SEM images of the vaporized nanodroplets and the generated microbubbles. (**F**) Temperature increases of mouse skin irradiated at various laser intensities (1, 2, 3, 4 W/cm^2^). NDs: nanodroplets.

To examine whether the synthesized nanodroplets could be vaporized by heating and used for cavitation-enhanced therapy, we first placed them in 60 °C water for 2 minutes. We observed the formation of gas bubbles on the bottle wall due to the vaporization of PFP nanodroplets (indicated by the yellow dashed rectangle in Figure 3A). By contrast, few gas bubbles were formed in 60 °C water when PFP was removed (Supplementary Figure 6). We then applied the 808-nm laser to heat the melanin nanoparticles and vaporize nanodroplets under the microscope. As shown in Figure 3B, when irradiated with a laser energy of 3 W/cm^2^, vaporization occurred and microbubbles emerged in the imaging view at 6 s post-irradiation (indicated by the white dashed rectangle). The microbubbles grew when laser irradiation was continued for another 10 s. We also quantified the vaporization time of nanodroplets irradiated at different laser intensities needed for them to grow into microbubbles with diameters larger than 10 µm. The vaporization time for laser intensities of 2, 3, and 4 W/cm^2^ was 22.16 ± 7.365, 18.87 ± 5.590, and 9.273 ± 4.427 s, respectively. However, as shown in Figure 3C, an intensity of 1 W/cm^2^ was insufficient to induce nanodroplet vaporization (indicated by the green x-shaped mark) during the 300-s-irradiation period.

The morphology of the vaporized nanodroplets was fixed using 2.5% glutaraldehyde and imaged using the standard SEM method. As shown in Figure 3D and 3E, melanin particles were released from the nanodroplets into the surrounding medium (indicated by the orange arrows) after vaporization. In addition, melanin nanoparticles accumulated around the shell of the microbubble (indicated by the yellow arrows). To examine the safety of *in vivo* application of 808-nm laser irradiation, the temperature of the mouse skin irradiated at different laser intensities was monitored. As shown in Figure 3F, after irradiation for 5 minutes at 1, 2, 3 and 4 W/cm^2^, the temperature was 36.27 ± 1.34, 42.80 ± 1.80, 57.63 ± 5.40 and 60.46 ± 3.43 °C, respectively. We note that thermal damage of the mouse skin was observed at laser intensities of 2, 3, and 4 W/cm^2^. An image of the mouse skin damaged by laser irradiation at 3 W/cm^2^ is given in Supplementary Figure 7.

### Safe and efficient nanodroplet vaporization using OASI

**Figure 4:**
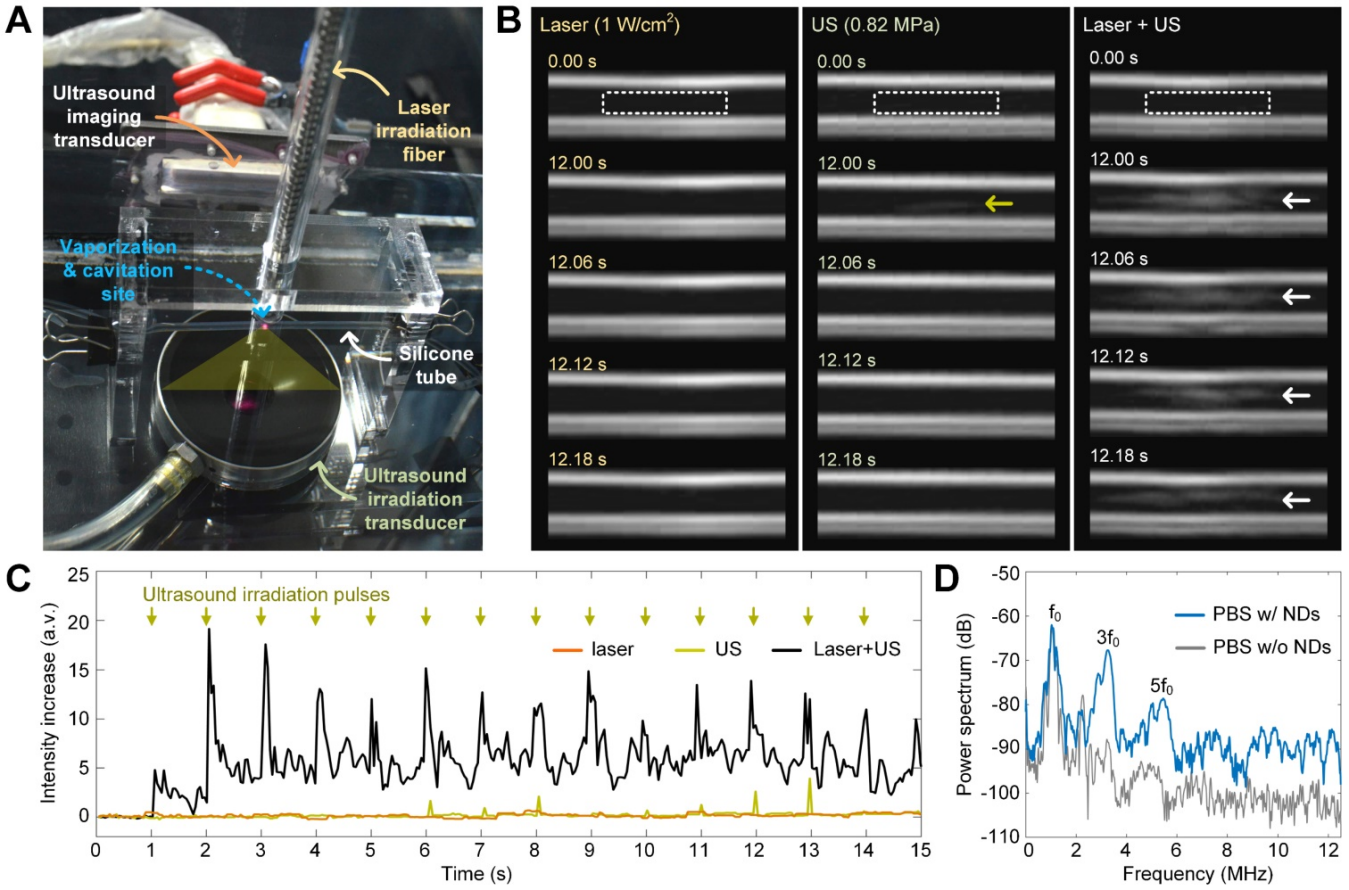
** Nanodroplet vaporization and microbubble cavitation induced by OASI.** (**A**) Experiment setup for real-time ultrasound imaging of nanodroplet vaporization inside a 1.5-mm-diameter silicone tube. (**B**) Ultrasound imaging sequences demonstrating the efficiency differences of nanodroplet vaporization induced by laser irradiation, ultrasound, or OASI. (**C**) Quantitative measurements of the intensity increases of echo signals reflected from the generated microbubbles. (**D**) Power spectra of the echo signals received with and without nanodroplets in PBS buffer during OASI. NDs: nanodroplets.

To reduce the laser intensity to a safe level for *in vivo* application (i.e., 1 W/cm^2^), we introduced ultrasound excitation for combined optical and acoustic nanodroplet vaporization. Laser irradiation can increase the temperature of melanin-cored nanodroplets, which reduces the ultrasound energy required for acoustic droplet vaporization 31. Therefore, we hypothesized that the combination of photothermal heating and ultrasound cavitation would reduce the thresholds of both to safe levels for efficient nanodroplet vaporization. To test this hypothesis, we aligned the laser beam and the ultrasound propagation path in the vertical direction and placed a silicone tube (1.5-mm inner diameter) containing the nanodroplet solution in the confocal region of the ultrasound and the laser beam. B-mode ultrasound imaging was also carried out in the horizontal direction to monitor nanodroplet vaporization and microbubble cavitation (Figure 4A).

The vaporization efficiencies of the nanodroplets irradiated by laser, ultrasound, and their combination at 26 °C are shown in Figure 4B. Consistent with the microscope observations, 1-W/cm^2^ laser irradiation alone failed to induce nanodroplet vaporization because there was no increase in the echo intensity in the ultrasound images, indicating that no microbubbles formed. Ultrasound irradiation alone (1.1-MHz frequency, 0.82-MPa peak negative pressure, 5-cycle pulse duration, and 1-Hz pulse repetition frequency) triggered the vaporization of some nanodroplets and resulted in a slight increase in the signal intensity in the ultrasound images (as indicated by the yellow arrow). By contrast, when the peak negative pressure of the ultrasound was increased to 0.82 MPa, the combined irradiation of laser and ultrasound induced significant vaporization of nanodroplets (as indicated by the white arrows in the ultrasound images). Quantitative measurements of the intensity increase of the echo signals due to nanodroplet vaporization and microbubble formation are shown in Figure 4C. As indicated by the green arrows, ultrasound excitation pulses were transmitted at a time interval of 1 s and the intensity of the echo signals increased with each ultrasound pulse. These results confirm the effectiveness of the combined laser and ultrasound irradiation method.

### Microbubble cavitation during laser- and ultrasound-induced vaporization

As demonstrated by the SEM imaging results in Figure 3D and the B-mode ultrasound images in Figure 4B, microbubbles were generated after nanodroplet vaporization. To examine the cavitation dynamics of the generated microbubbles driven by pulsed ultrasound irradiation, we replaced the ultrasound imaging transducer with an ultrasound echo-recording transducer, which passively received the acoustic signals reflected by the microbubbles. Representative power spectra of the received signals are shown in Figure 4D. Compared with the signal from a blank solution without nanodroplets, the signal from the nanodroplet solution had an increased power intensity of the harmonic frequencies (*f*_0_, 3*f*_0_, and 5*f*_0_,) and broadband noise. We note that the harmonics demonstrate the formation and oscillation of the microbubbles, whereas the broadband noise represents the collapse of the microbubbles 32. Therefore, we confirmed that nanodroplet vaporization and these two physical effects of microbubble cavitation are induced in our proposed sono-chemo-photothermal therapy for mechanically facilitating melanin and DOX penetration into tumors.

### Enhanced intratumoral accumulation of melanin and mechanical breaking of the tumor tissue

**Figure 5:**
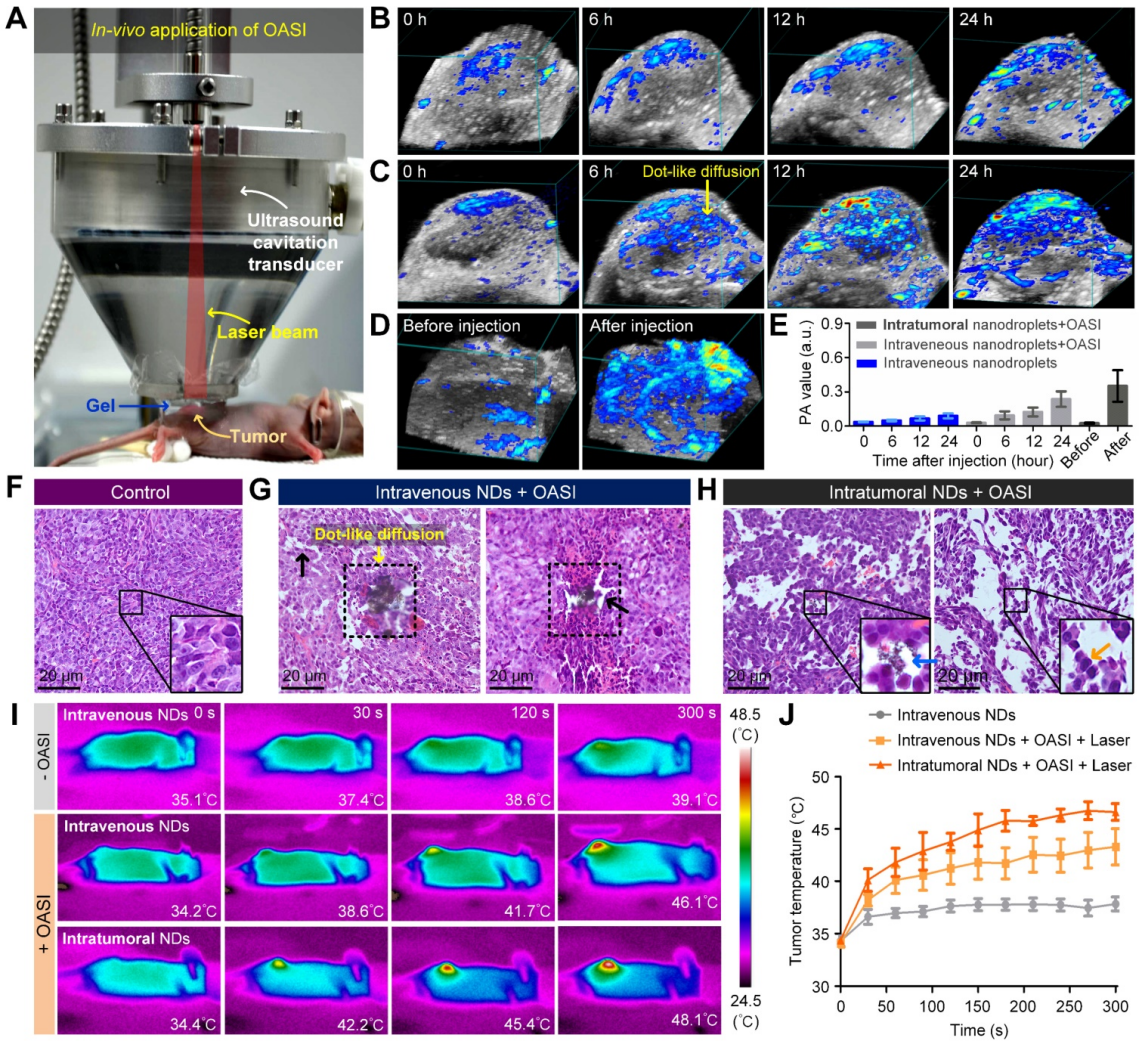
** Intratumoral accumulation of melanin and the mechanical breaking of tumor tissue.** (**A**) Experimental setup for *in vivo* application of OASI. (**B-D**) 3D photoacoustic images showing the efficiency differences of melanin penetration and accumulation inside tumors treated (B) without OASI, (C) with OASI in an intravenous manner, and (D) with OASI in an intratumoral manner. (**E**) Quantitative measurements of the photoacoustic intensities of the images shown in (B-D). (**F-H**) Representative H&E staining results of tumors in the control group (F), the intravenous NDs group with OASI (G), and the intratumoral NDs group with OASI (H). (**I**) Melanin accumulation inside the tumors enhanced by the photothermal effect of laser irradiation at 1 W/cm^2^. (**J**) Quantitative measurements of the temperature increases shown in (I).

To examine whether the mechanical effects induced by nanodroplet vaporization and microbubble cavitation can break diffusion barriers and increase melanin penetration, the intratumoral melanin distribution was monitored using 3D photoacoustic imaging at various timepoints after nanodroplet injection. For the control group, nanodroplets were intravenously injected into the mice (*n* = 3) without any treatment. For both the intravenously-injected nanodroplet group (*n* = 3) and the intratumorally-injected nanodroplet group (*n* = 3), 2-minute combined irradiation with the laser (1 W/cm^2^) and ultrasound (1.1-MHz frequency, 0.82-MPa peak negative pressure, 5-cycle pulse duration, and 1-Hz pulse repetition frequency) was carried out immediately after nanodroplet injection using the experiment setup shown in Figure 5A. The 3D photoacoustic imaging results in Figure 5B show that, without nanodroplet vaporization and microbubble cavitation, melanin only slightly accumulated in the periphery of the tumor region after 24-hour passive diffusion. By contrast, when the intravenously-injected nanodroplets were activated by OASI, dot-like diffusion of melanin emerged in the whole region of the tumor, breaking the tumor blood vessels and tumor cells (Figure 5C). For the intratumorally-injected nanodroplet group, the melanin distribution was much more homogenous inside the tumors (Figure 5D). Quantitative measurements (Figure 5E) show that, compared with the control group, the photoacoustic value of the tumor increased 2.64- and 3.90-fold in the intravenous and intratumoral injection groups, respectively.

To examine whether the accumulated melanin could enhance the efficacy of photothermal therapy for tumors, mice were randomly divided into the following three groups and exposed to laser irradiation for 5 minutes at 1 W/cm^2^: an intravenous nanodroplet group without OASI (*n* = 3), an intravenous nanodroplet group (*n* = 3) 24 hours after OASI, and an intratumoral nanodroplet group (*n* = 3) 5 minutes after OASI. Photothermal imaging results of the increase in tumor temperature during laser exposure are given in Figure 5I. The temperature of the tumor region after laser exposure was measured to be 37.83 ± 1.14 °C (intravenous nanodroplet group without OASI), 43.28 ± 4.25 °C (intravenous nanodroplet group with OASI), and 46.63 ± 1.41 °C (intratumoral nanodroplet group with OASI) (Figure 5J). These results suggest that the nanodroplets combined with OASI can enhance melanin accumulation and promote photothermal therapy. We note that the calculated power intensity (*I*_SPTA_) of ultrasound irradiation in OASI treatment was 0.022 mW/cm^2^, at which the thermal effect is negligible compared with laser irradiation at 1 W/cm^2^. Supplementary Figure 9 demonstrates that ultrasound irradiation had no effect on melanin-mediated photothermal therapy.

### Release and accumulation of DOX

**Figure 6:**
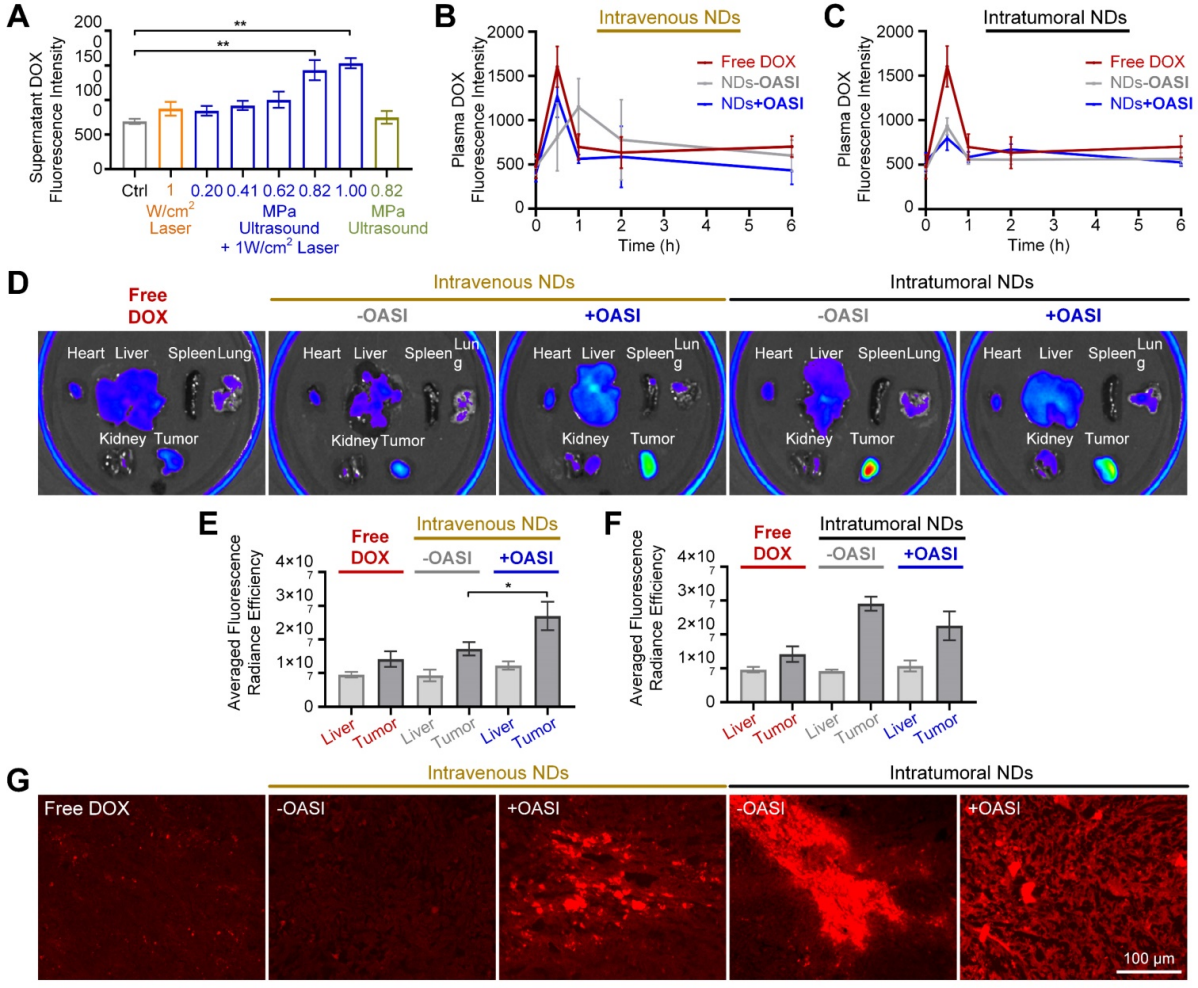
** Release and accumulation of DOX.** (**A**) *In vitro* release efficiency of DOX from nanodroplets exposed to laser-only, ultrasound-only, or OASI treatment. (**B,C**) Pharmacokinetics of DOX in the blood of mice treated with free DOX, intravenous nanodroplet injection with and without OASI (B), and intratumoral nanodroplet injection with and without OASI (C). (**D**) *Ex vivo* fluorescence images of major organs and tumors of mice treated according to the different experiment protocols. (**E,F**) Statistical results showing the distribution of DOX in tumors and livers of mice of treated with free DOX, intravenous nanodroplet injection with and without OASI (E), and intratumoral nanodroplet injection with and without OASI (F). (**G**) Fluorescence imaging results of DOX on the tumor slides of the different experimental groups. NDs: nanodroplets. DOX: doxorubicin.

As shown in Figure 6A, the *in vitro* release efficiency of DOX from the PVA shells of the nanodroplets was studied using different irradiation parameters. We found that laser-only or ultrasound-only irradiation only slightly induced DOX release. By contrast, when the peak negative pressure of the ultrasound was increased to 0.82 MPa, OASI treatment induced significant DOX release (*p* < 0.01). We also studied the pharmacokinetics of the released DOX in blood. As shown in Figure 6B, when the nanodroplets were intravenously injected into the mice, DOX was released from the nanodroplets by OASI treatment in the tumor region. Without OASI treatment, DOX leaked from the nanodroplets within 2 hours owing to blood circulation. When the nanodroplets were intratumorally injected, no significant difference was found in the DOX pharmacokinetics between the group treated with OASI and that without OASI (Figure 6C).

We used *ex vivo* fluorescence imaging to study the DOX distribution in tumor-bearing mice 24 hours after free DOX injection, intravenous nanodroplet injection treated with and without OASI, and intratumoral nanodroplet injection treated with and without OASI (Figure 6D). For the mice injected with free DOX, the averaged fluorescence radiance intensity of DOX in the tumors was slightly higher than that in the livers (Figure 6E). However, for the mice that received intravenous nanodroplet injection, DOX accumulated in a higher concentration in the tumors. Moreover, OASI treatment significantly increased DOX accumulation in tumors (*p* < 0.05). Notably, for the mice that received intratumoral nanodroplet injection, OASI treatment decreased the concentration of DOX in the tumor regions (Figure 6F). Furthermore, we studied the intratumoral distribution of DOX on tumor slides for the different experimental groups. This further confirmed that OASI treatment increased DOX accumulation in the tumors of the mice that received intravenous nanodroplet injection. We also found that for the mice that received intratumoral nanodroplet injection and were treated with OASI, the distribution of DOX was more homogenous than for the other groups, and the interstitial space of the tumors was mechanically enlarged.

### *In vitro* synergistic effect of secondary chemo-photothermal therapy

**Figure 7:**
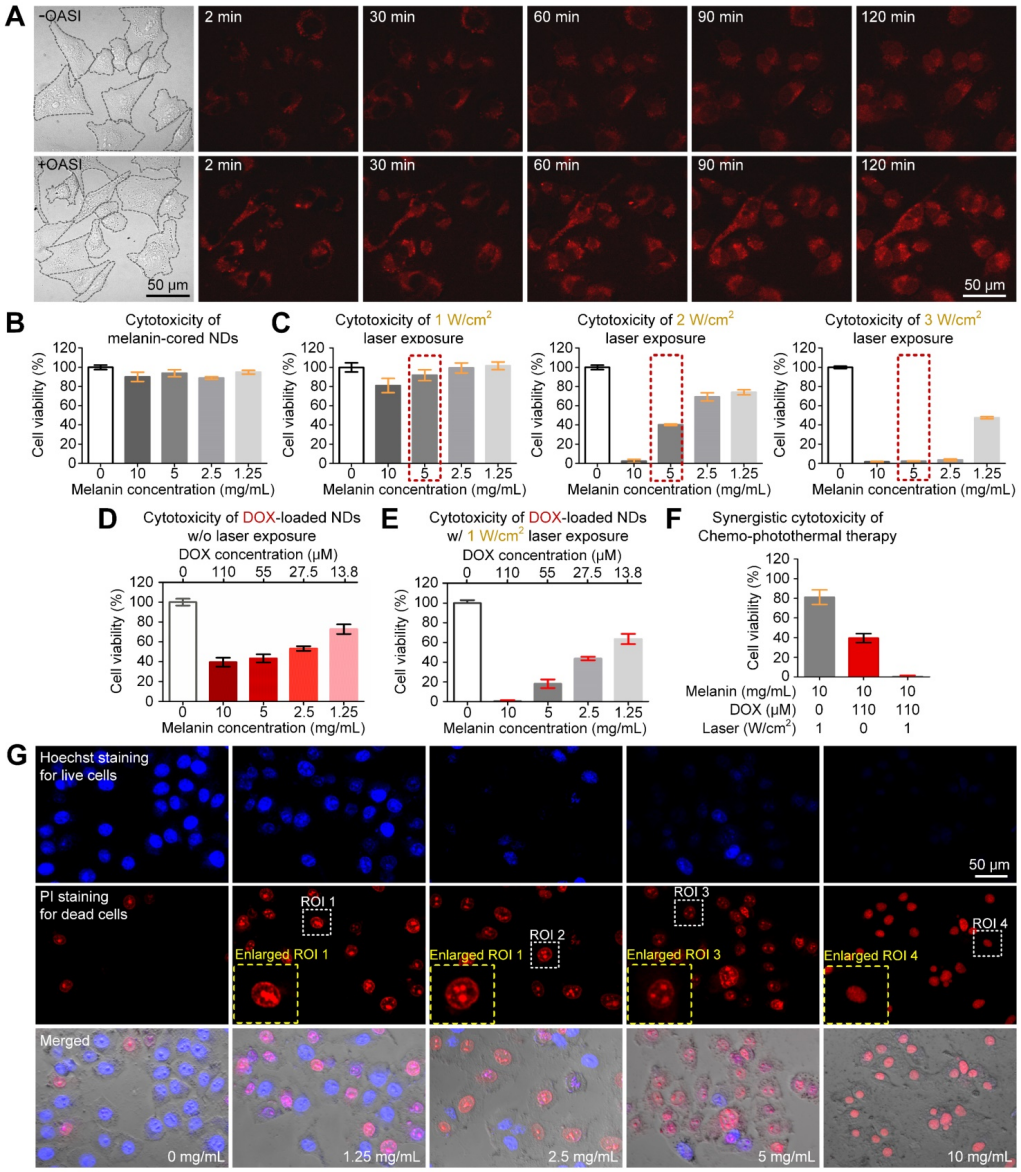
***In vitro* synergistic anti-tumor effect of secondary chemo-photothermal therapy.** (**A**) Time-lapsed confocal microscope images demonstrating the difference in cellular uptake efficiency of DOX with and without OASI treatment. (**B,C**) Cytotoxicity of melanin-cored nanodroplets without and with 1-W/cm^2^ laser irradiation, respectively. (**D,E**) Cytotoxicity of melanin-cored and DOX-loaded nanodroplets without and with 1-W/cm^2^ laser irradiation, respectively. (**F**) Synergistic effect of secondary chemo-photothermal therapy. (**G**) Representative microscope images demonstrating the cytotoxicity of the melanin- and DOX-mediated chemo-photothermal therapy. DOX: doxorubicin.

We examined the effect of OASI on the cellular uptake of DOX using real-time confocal imaging. As shown in Figure 7A, intracellular distribution of DOX increased immediately after OASI treatment in contrast with the untreated group. Moreover, at the end of the observation, intracellular concentration of DOX in the OASI group was much higher. As shown in Figure 7B, the melanin-cored nanodroplets without DOX loading did not exhibit discernible cytotoxicity, even at a relatively high concentration (10 mg/mL), demonstrating the good biocompatibility of this nanoplatform. To examine the photothermal therapeutic effect of the melanin-cored nanodroplets without DOX loading, the cells were incubated with the nanodroplet medium of different concentrations of melanin nanoparticles (1.25, 2.5, 5, 10 mg/mL). When laser irradiation was applied, the cytotoxicity of the nanodroplets increased with laser intensity (Figure 7C). The viability of cells cultured in the nanodroplet medium containing 5 mg/mL melanin (indicated by the red dashed rectangle) after photothermal therapy at 1, 2, and 3 W/cm^2^ was 91.85 ± 9.91%, 40.17 ± 1.30%, and 0.72 ± 2.59%, respectively.

To examine the chemotherapeutic efficiency of the DOX-loaded nanodroplets, the cells were incubated with 1.25, 2.5, 5, or 10 mg/mL melanin nanoparticles and 13.8, 27.5, 55, or 110 μM DOX, respectively. As shown in Figure 7D, the nanodroplet media containing higher concentrations of DOX resulted in more pronounced cell death. Even with the highest DOX concentration (110 μM), the cells had a viability of 39.54 ± 7.836%. To investigate the synergistic effect of combined photothermal and chemotherapy, the cells were incubated with nanoparticles with DOX loading and under laser irradiation at 1 W/cm^2^ (Figure 7E). Compared with the anti-tumor efficiency of chemotherapy (81.08 ± 12.92%) or photothermal therapy (39.54 ± 7.84%), the therapeutic efficiency of the combined chemo-photothermal method was greatly improved (0.45 ± 2.00%), confirming the synergistic effect of this method (Figure 7F). In Figure 7G, we further found that, when the melanin concentration was increased to 10 mg/mL, the combined chemo-photothermal therapy induced the condensation of the cell nuclei (indicated by the blue dashed rectangle). The calculated IC50 value of free DOX was 37.62 µM. The calculated IC50 value of DOX-loaded nanodroplets was 2.13 mg/mL. According to the formula reported by Zhao et al. 33, the calculated combination index for chemotherapy and photothermal therapy was 0.43.

### *In vivo* therapeutic effect of sono-chemo-photothermal therapy

**Figure 8:**
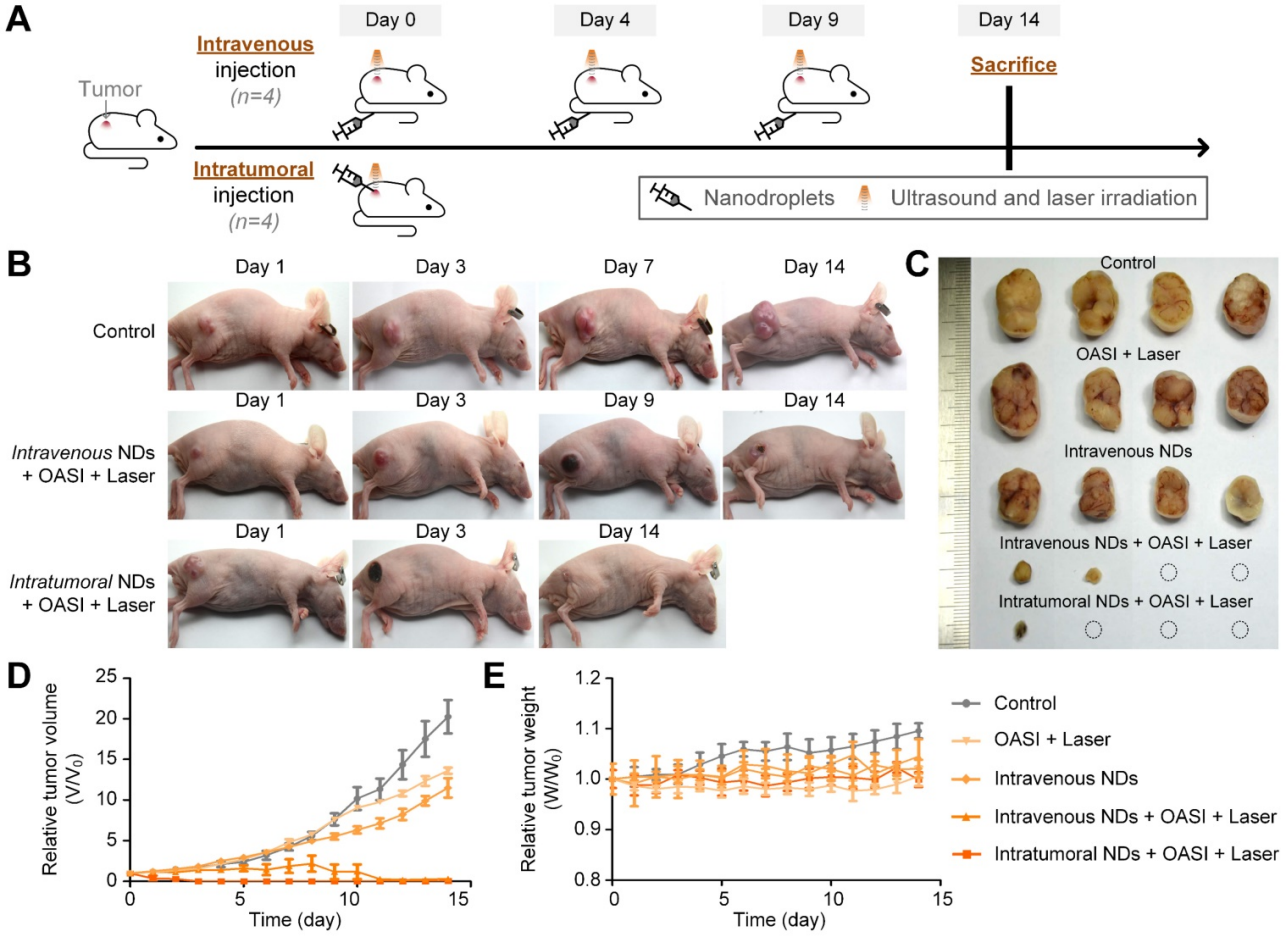
***In vivo* antitumor effect of the proposed sono-chemo-photothermal therapy.** (**A**) Schematic illustration of the sono-chemo-photothermal therapy carried out with either intravenous or intratumoral injection. (**B**) Representative images of mice bearing melanoma tumors after different kinds of treatment. (**C**) Tumors removed from the sacrificed mice after a 14-day observation period. (**D**) Inhibition of tumor growth. (**E**) Measured body weight.

The tumor-bearing mice were randomly divided into five groups (four mice in each group): a control group without any treatment (group 1); an irradiation-only (with OASI and subsequent laser treatment) group without nanodroplet injection (group 2); an intravenously injected nanodroplet group without irradiation (group 3); an intravenously injected nanodroplet group with OASI and laser irradiation (group 4); and an intratumorally injected nanodroplet group with OASI and laser irradiation (group 5). As shown in Figure 8A, OASI and laser irradiation were carried out three times (*n* = 3) with an interval of four days in groups 2 and 4. By contrast, OASI and laser irradiation were performed only once in group 5 on day 3, at which point a significant anti-tumor effect had already been achieved (Figure 8B). As shown in Figure 8C and 8D, tumors in group 1 exhibited rapid growth. Monomodal and bimodal therapy carried out with irradiation only (group 2) and the nanodroplet-only group without irradiation (group 3) showed mild anti-tumor effects. Sono-chemo-photothermal therapy carried out in group 4 required three repetitions to elicit a significant anti-tumor effect. By contrast, sono-chemo-photothermal therapy carried out by intratumoral injection (group 5) resulted in an efficient anti-tumor effect, with the tumors being eliminated on day 3. No measurable loss in body weight was recorded in any of the groups (Figure 8E). In Supplementary Figure 10, we further confirmed that sonotherapy and sono-chemotherapy slowed down tumor growth, whereas sono-chemo-photothermal therapy fully eliminated the tumors.

**Figure 9:**
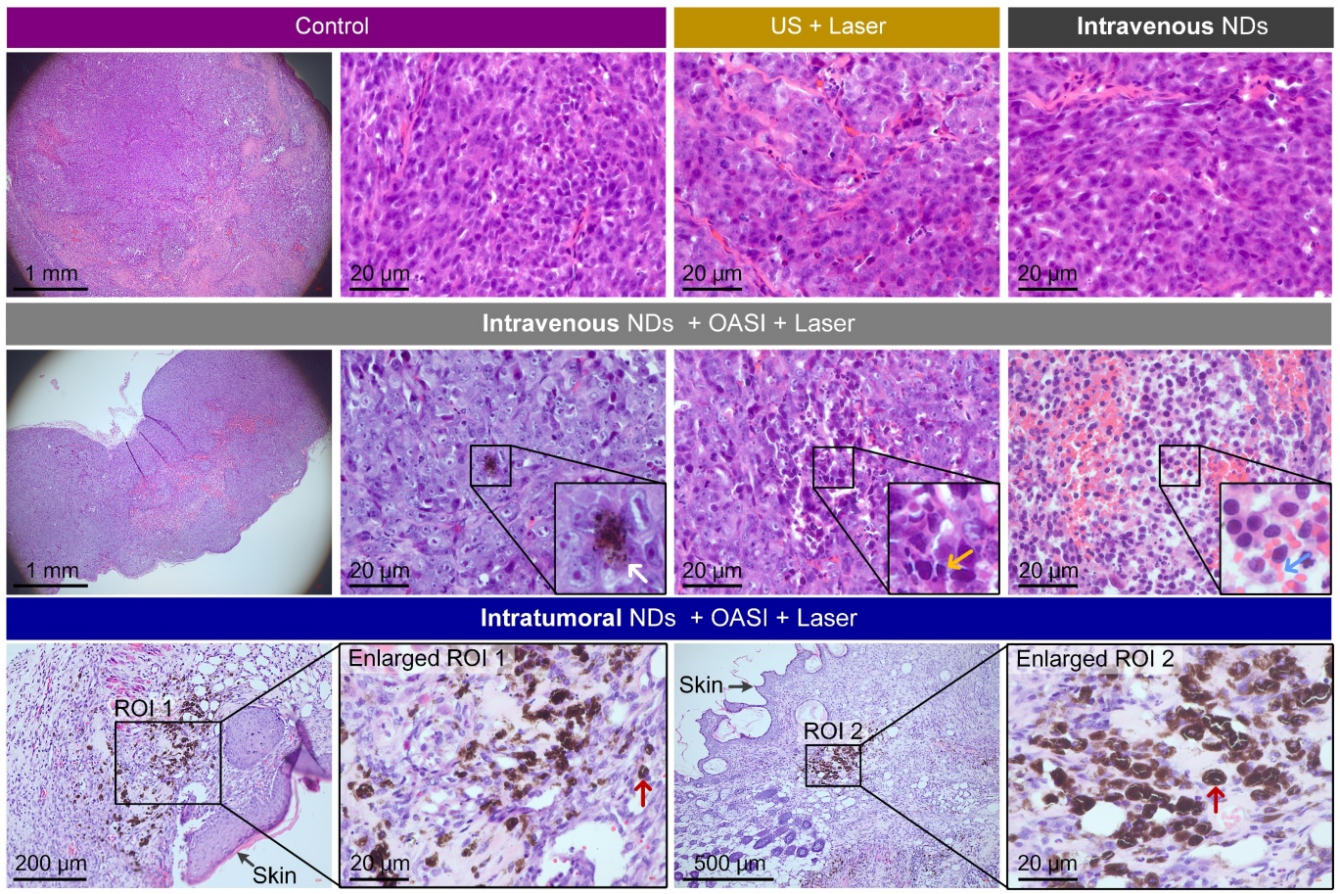
Representative H&E staining images showing the histological structure of tumors exposed to the different types.

**Figure 10:**
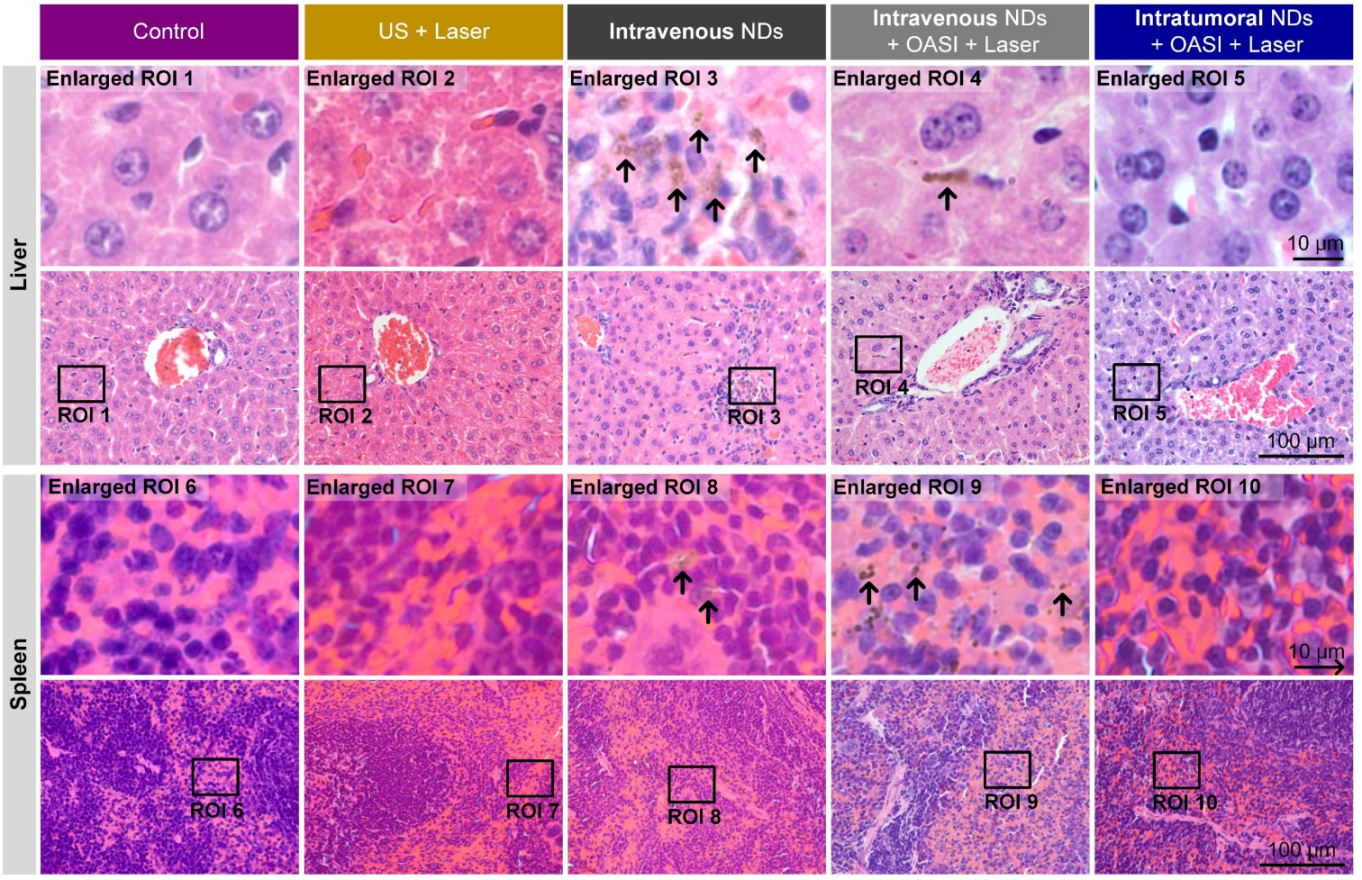
Representative H&E staining images showing the accumulation of melanin in the livers and spleens of mice subjected to the different treatments.

From the H&E staining results shown in Figure 9, no obvious damage of the tumor tissue structure or tumor cell morphology was found in groups 1-3. Consistent with the results shown in Figure 5, we observed the dot-like accumulation of melanin (indicated by the white arrow) in group 4. In particular, nucleus fragmentation of the tumor cells (indicated by the yellow arrow) and the presence of abundant blood cells demonstrating inflammation (indicated by the blue arrow) were also observed in the histological examination of the tumors in group 4. In group 5, melanin mainly accumulated in the original tumor region underneath the skin. More interestingly, we found that the intratumorally-injected melanin was engulfed by, and accumulated within, these cells, as indicated by the red arrows. As shown in Figure 10, melanin was found in the livers and spleens of mice in groups 3 and 4 (indicated by the black arrows). By contrast, no melanin was found in the major organs of mice in group 5. Melanin was not found in the hearts, lungs, or kidneys of the mice in any of the five groups (Supplementary Figure 11). We also carried out complete blood count and blood biochemistry analyses to examine the *in vivo* toxicity of the nanodroplets. As shown in Supplementary Figures 12 and 13, no obvious changes were observed in the mice injected with melanin-cored nanodroplets, confirming the biocompatibility of the nanodroplets. However, when DOX was loaded on the PVA shells of the nanodroplets, the serum levels of alanine aminotransferase and triglyceride significantly changed in the mice intravenously injected with 200 µL nanodroplet solution.

## Discussion

Although photothermal therapy is a promising cancer treatment modality, its clinical application faces two challenges: (i) the biocompatibility of photothermal agents (e.g., gold nanoparticles) is relatively poor, causing persistent cytotoxicity to the liver and brain 34, 35; and (ii) the accumulation efficiency of photothermal agents varies greatly among tumors of different patients, and among tumors of different types and sizes, therefore leading to inconsistent therapeutic outcomes 36. Our melanin-cored nanodroplets and delivery strategy overcome these two challenges because the photothermal agent, natural melanin, has good biocompatibility, and the mechanical forces generated by nanodroplet vaporization increase blood vessel permeability and the efficiency of melanin accumulation.

For laser irradiation alone, a high laser intensity (>2 W/cm^2^) was required to efficiently heat the melanin, which resulted in obvious skin damage. For safe and efficient nanodroplet vaporization, our combined ultrasound and laser irradiation method uses a laser intensity of 1 W/cm^2^ and ultrasound mechanical index of 0.78. Real-time ultrasound imaging of nanodroplet vaporization confirmed that the introduction of ultrasound not only decreased the energy threshold of laser irradiation for nanodroplet vaporization, but also triggered the cavitation of the microbubbles generated by nanodroplet vaporization. The mechanical effects exerted by nanodroplet vaporization and microbubble cavitation are conducive to cancer therapy by increasing melanin penetration and accumulation (as seen in the photoacoustic imaging results) and by directly breaking the tumor tissue and cell membrane (as seen in the H&E staining results). Thermal imaging revealed that the temperature of the tumors increased to 43.28 ± 4.25 °C and 46.63 ± 1.41 °C after subsequent laser-mediated photothermal therapy for the intravenously-injected and intratumorally-injected nanodroplet groups, respectively.

Consistent with a previous study, which showed that the efficiency of photothermal therapy is positively correlated with the laser intensity and concentration of photothermal agents 37, we found that laser irradiation at 2 and 3 W/cm^2^ killed all tumor cells in the presence of 10 mg/mL melanin, whereas lower-intensity (1 W/cm^2^) irradiation only reduced the cell viability by ~20%. To fully kill the A375 melanoma cells, we further loaded the anti-cancer drug DOX onto the PVA shells of the melanin-cored nanodroplets and confirmed the synergistic therapeutic effect of DOX and melanin *in vitro*. In this way, our melanin-cored and doxorubicin-loaded nanodroplets combined the mechanical wounding effects of the ultrasound contrast agent, the cytotoxic effects of the chemotherapy drug, and the photothermal effects of melanin for synergistic sono-chemo-photothermal therapy. After intravenous nanodroplet injection and OASI treatment,* ex vivo* fluorescence imaging showed that only 2.247 ± 0.286 μM DOX had accumulated in the melanoma tumor region; in this case, three cycles of treatment were required to induce a significant anti-tumor effect. By contrast, the local concentration of DOX in the tumor regions after intratumoral nanodroplet injection was 27 μM, with only a single round of therapy being required to eliminate the tumor. The difference in the therapeutic efficiencies of these two injection methods is due to the greater accumulation of melanin and DOX in the tumor region for the intratumoral injection group.

In this study, we used the same ultrasound pulse setting for nanodroplet vaporization and microbubble cavitation because the electrical waves for each cannot be separately edited with our equipment. Future work should be carried out to investigate whether different peak negative pressures would induce different modes of blood vessel leakage. For example, direct observation by two-photon fluorescence microscopy revealed that fast leakage of blood vessels occurred at all ultrasound pressures, whereas slow and sustained leakage was more frequently induced by low-pressure ultrasound 38. Moreover, Samiotaki et al. showed that opening of the blood-brain barrier induced by microbubbles and 0.3-MPa ultrasound lasted for less than three days, whereas that induced by microbubbles and 0.6-MPa ultrasound persisted for more than five days 39. Therefore, greater effort should be placed on examining the recovery dynamics of vaporization and cavitation-wounded blood vessels to optimize the ultrasound parameters and time intervals required for repeated intravenous injection and chemo-photothermal therapy.

We note that ultrasound cavitation can exert an immune response for tumor therapy in two ways: (i) by inducing a sterile inflammatory response by increasing the production of cytokines (e.g., IL-1,IL-18, and TNFα) and the infiltration of CD68+ macrophages 40; and (ii) by increasing immunogenic presentation of tumor antigens to T cells by wounding the plasma membrane of tumor cells 41. Moreover, chemo-photothermal therapy can also elicit anti-tumor immunity by upregulating dendritic cells in tumor-draining lymph nodes and inducing a robust CD8+ T cell response in tumors 42. In future work, the potential of our method for immunomodulation and suppression of tumor metastasis should be explored.

## Conclusion

In summary, we designed and synthesized self-assembling perfluoropentane nanodroplets with good biocompatibility for cancer theranostics. Natural melanin was encapsulated into the perfluorocarbon cores of the nanodroplets for photothermal heating and DOX was loaded onto their PVA shells for chemotherapy. We used synergistic opto-acoustic irradiation to safely vaporize the nanodroplets and to cavitate the microbubbles generated by vaporization. During irradiation, the mechanical effects of nanodroplet vaporization and microbubble cavitation directly broke intratumoral diffusion barriers as well as the tumor cell membrane to increase the penetration and accumulation of melanin and DOX. Using secondary laser irradiation, melanin-mediated photothermal therapy and DOX-mediated chemotherapy further synergistically killed melanoma cells in both *in vitro* and *in vivo* experiments. Thus, we have demonstrated the great potential of our natural melanin-cored and DOX-loaded nanoparticles for effective sono-chemo-photothermal cancer therapy.

## Supplementary Material

Supplementary figures and tables.
