# Supplementary material for: Opto-acoustic synergistic irradiation for vaporization of natural melanin-cored nanodroplets at safe energy levels and efficient sono-chemo-photothermal cancer therapy
Source: Theranostics. 2020 Aug 20;10(23):10448–65. doi: 10.7150/thno.44879 (PMC7482808; doi:10.7150/thno.44879)
Supplement: Supplementary file 1 — Supplementary figures and tables. [file thnov10p10448s1.pdf]

Supporting Information: **Opto-acoustic synergistic irradiation for vaporization of natural melanin-cored nanodroplets at safe energy levels and efficient sono-chemo-photothermal cancer therapy**

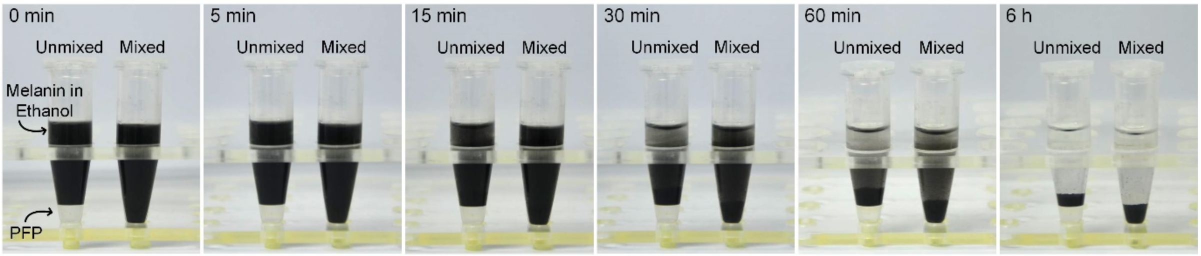

Supplementary Figure 1. Time-lapsed images demonstrating the sedimentation process of melanin nanoparticles in ethanol (Unmixed) and the ethanol-PFP mixture (Mixed). PFP: perfluoropentane.

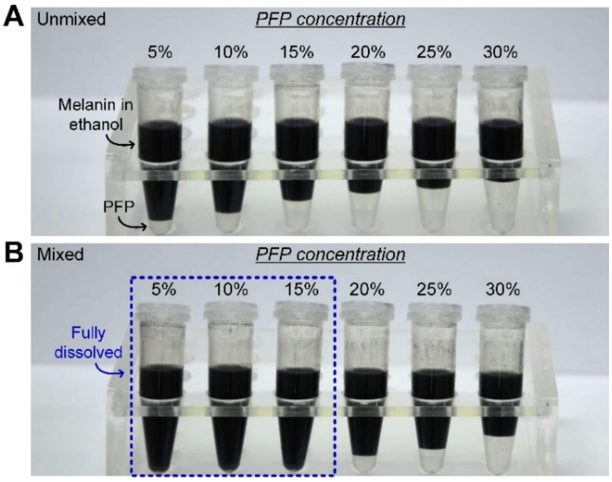

Supplementary Figure 2. Images showing the solubility of PFP of different volume ratios (5%-30%) in ethanol before (A) and after (B) mixing by agitation. PFP: perfluoropentane.

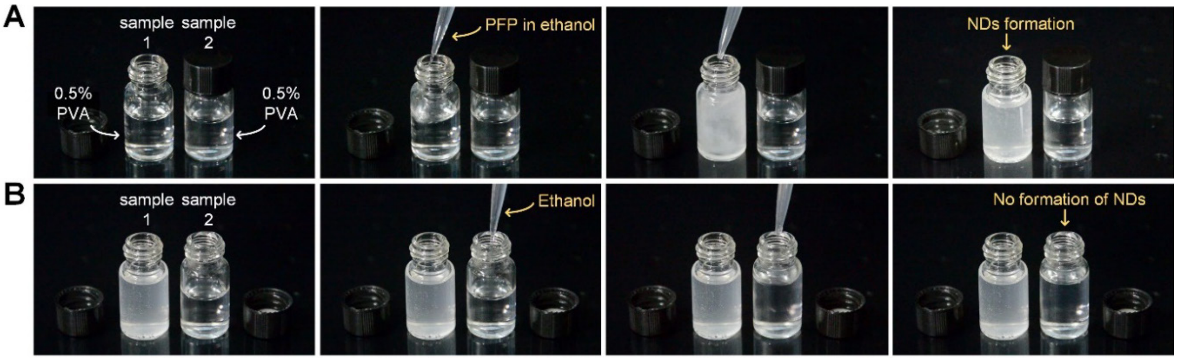

Supplementary Figure 3. Time-lapsed images showing the emulsification process of the nanodroplets by the ouzo effect. (A) The addition of PFP and ethanol to the aqueous phase resulted in nanodroplet formation. (B) The addition of ethanol alone failed to produce nanodroplets. NDs: nanodroplets. PFP: perfluoropentane.

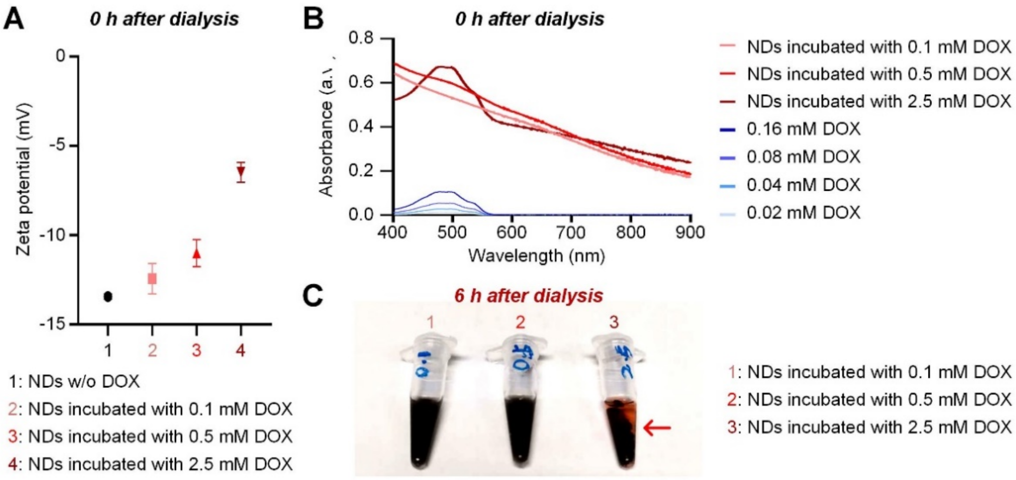

Supplementary Figure 4. Characterization of the nanodroplets loaded with different amounts of DOX. Zeta potential (A) and absorption spectra (B) of nanodroplets incubated with 0.1, 0.5, and 2.5 mM DOX solution during the drug-loading procedure. (C) Photographs of nanodroplet solutions showing the aggregation of nanodroplets and the leakage of DOX in group 3. The values are presented as the mean ± standard error of the mean. NDs: nanodroplets. DOX: doxorubicin.

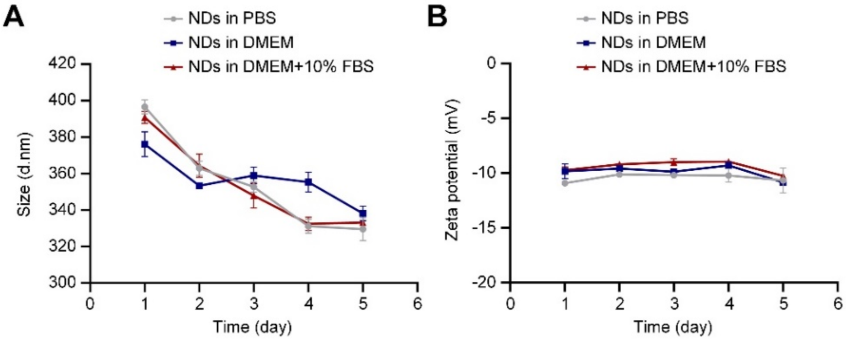

Supplementary Figure 5. Nanodroplet stability. Size (A) and zeta potential (B) measurements of the nanodroplets in PBS buffer, DMEM medium, and DMEM medium supplemented with 10% FBS. The values are presented as the mean ± standard error of the mean. NDs: nanodroplets.

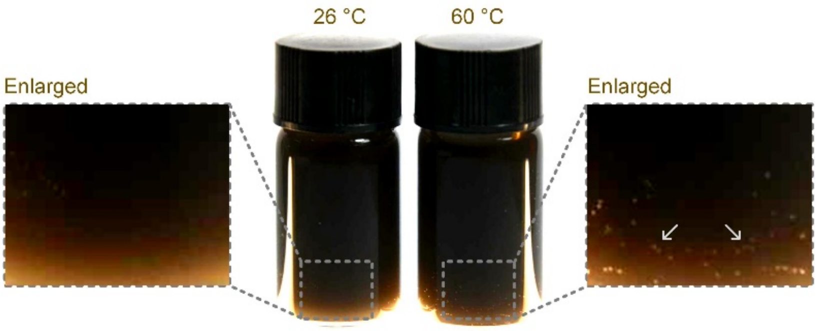

Supplementary Figure 6. Image showing that few gas bubbles were formed in 60 °C water when PFP was removed from the nanodroplets.

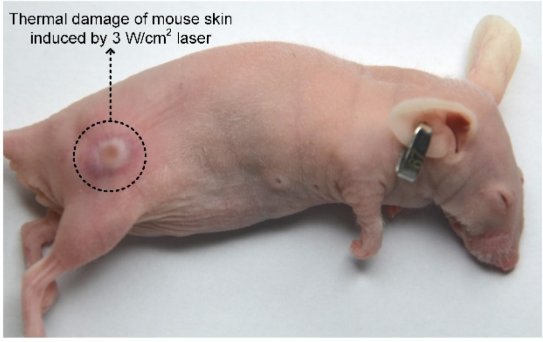

Supplementary Figure 7. Representative image showing thermal damage of the mouse skin induced by laser irradiation at 3 W/cm^2 for 5 minutes.

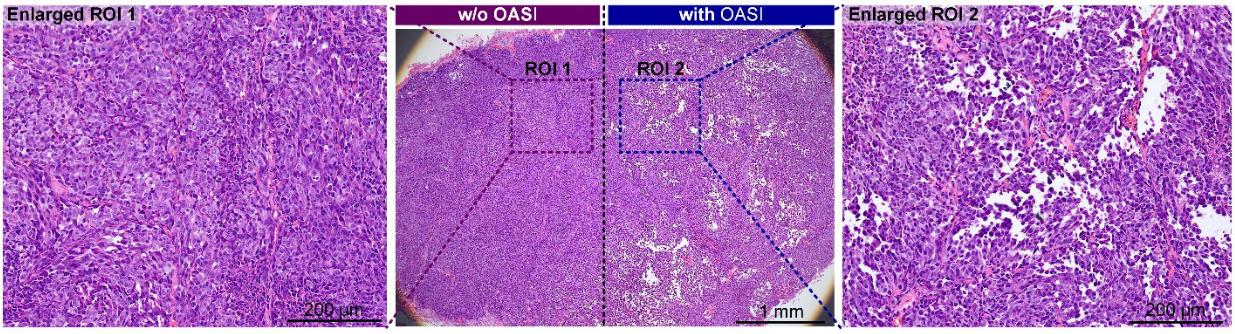

Supplementary Figure 8. H&E-stained images of the tumor demonstrating the spatial resolution of the mechanical breaking effects induced by a single OASI treatment. ROI: region of interest.

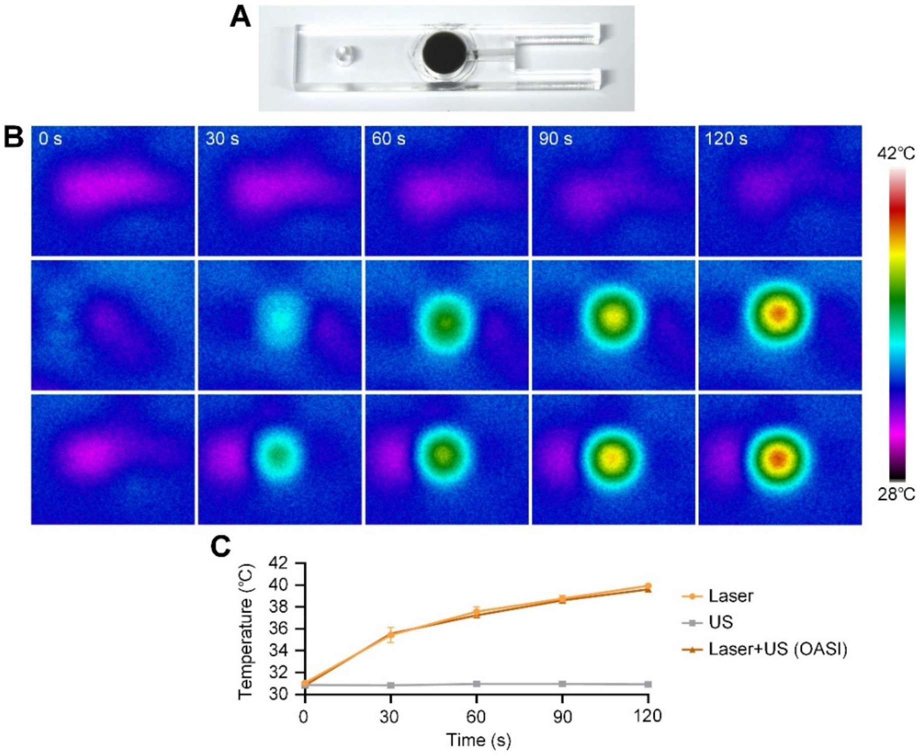

Supplementary Figure 9. *In vitro* photothermal performance of ultrasound, laser, and OASI irradiation. (A) Immersible sample chamber used for the different treatments. (B) Time-lapsed photothermal images demonstrating the temperature changes of the nanodroplets during 2-minute ultrasound, laser, and OASI

irradiation. (C) Temperature elevation measured during 2-minute ultrasound, laser, and OASI irradiation. The values are presented as the mean ± standard error of the mean.

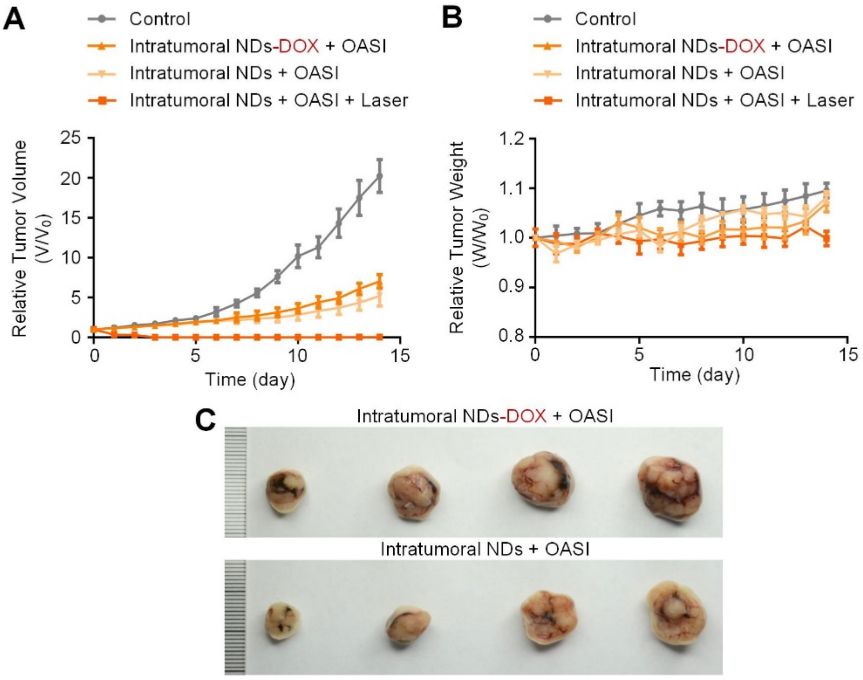

Supplementary Figure 10. *In vivo* antitumor sonotherapy and sono-chemo therapy. (A) Inhibition of tumor growth. (B) Measured body weights (B). (C) Tumors removed from the sacrificed mice after a 14-day observation duration. The values are presented as the mean ± standard error of the mean. NDs: nanodroplets. DOX: doxorubicin.

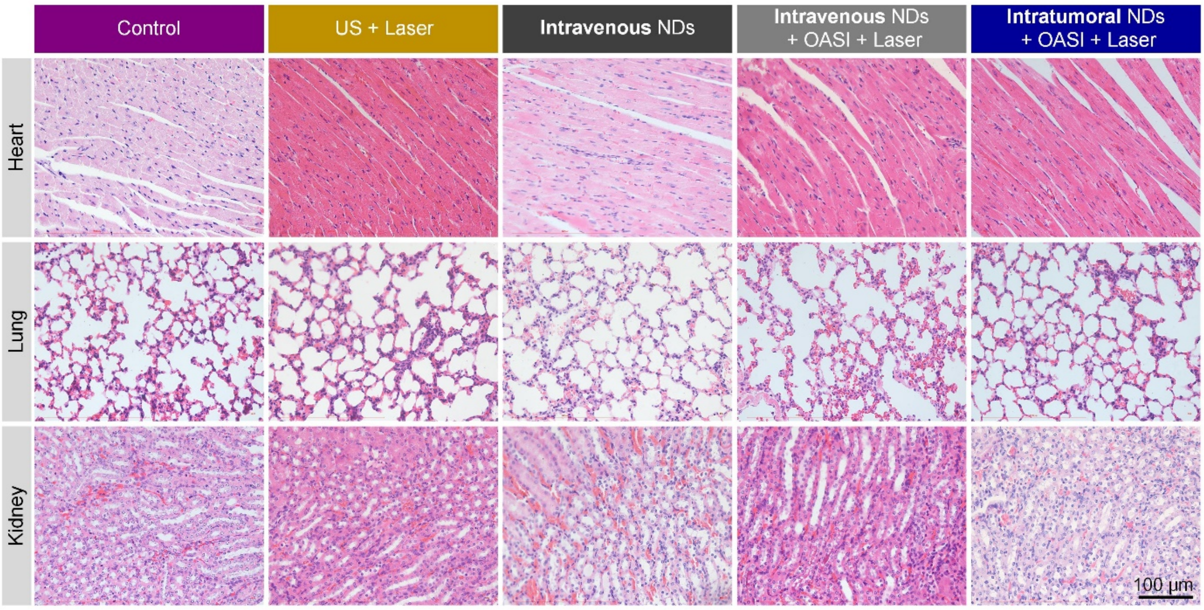

Supplementary Figure 11. Representative H&E-stained images of the hearts, lungs, and kidneys of mice treated using the different experimental protocols. NDs: nanodroplets.

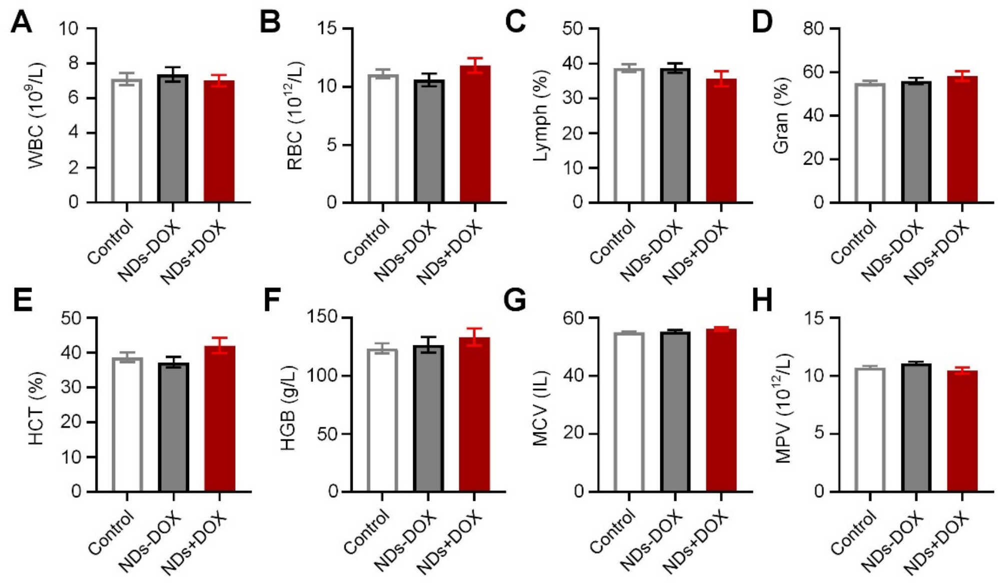

Supplementary Figure 12. Complete blood count of mice 7 days after injection of PBS (Control), and nanodroplets with (NDs+DOX) and without (NDs-DOX) DOX-loading. (A) Number of white blood cells (WBC). (B) Number of red blood cells (RBC). (C) Percentage of lymphocytes (Lymph). (D) Percentage of granulocytes (Grun). (E) Hematocrit (HCT). (F) Hemoglobin (HGB). (G) Mean corpuscular volume (MCV). (H) Mean platelet volume (MPV). The values are presented as the mean ± standard error of the mean. NDs: nanodroplets. DOX: doxorubicin.

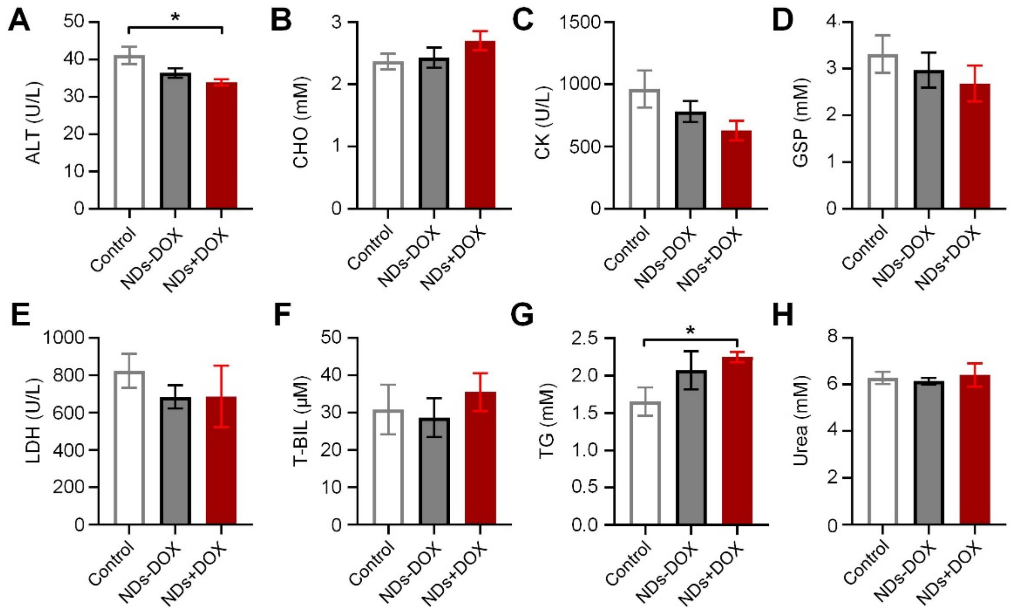

Supplementary Figure 13. Blood biochemistry of mice 7 days after injection of PBS (Control), and nanodroplets with (NDs+DOX) and without (NDs-DOX) DOX-loading. (A) Alanine aminotransferase (ATL). (B) Cholesterol (CHO). (C) Creatine kinase (CK). (D) Glycosylated serum protein (GSP). (E) Lactate dehydrogenase (LDH). (F) Total bilirubin (T-BIL). (G) Triglyceride (TG). (H) Urea. The values are presented as the mean ± standard error

of the mean. NDs: nanodroplets. DOX: doxorubicin.
